# Semi-automatic tracking, smoothing and segmentation of hyoid bone motion from videofluoroscopic swallowing study

**DOI:** 10.1371/journal.pone.0188684

**Published:** 2017-11-28

**Authors:** Won-Seok Kim, Pengcheng Zeng, Jian Qing Shi, Youngjo Lee, Nam-Jong Paik

**Affiliations:** 1 Department of Rehabilitation Medicine, Seoul National University College of Medicine, Seoul National University Bundang Hospital, Seongnam-si, Gyeonggi-do, Korea; 2 School of Mathematics & Statistics, University of Newcastle, UK; 3 Data Science for Knowledge Creation Research Center, Seoul National University, Seoul, Korea; 4 Department of Statistics, Seoul National University, Seoul, Korea; Ecole Nationale d'Ingenieurs de Sfax, TUNISIA

## Abstract

Motion analysis of the hyoid bone via videofluoroscopic study has been used in clinical research, but the classical manual tracking method is generally labor intensive and time consuming. Although some automatic tracking methods have been developed, masked points could not be tracked and smoothing and segmentation, which are necessary for functional motion analysis prior to registration, were not provided by the previous software. We developed software to track the hyoid bone motion semi-automatically. It works even in the situation where the hyoid bone is masked by the mandible and has been validated in dysphagia patients with stroke. In addition, we added the function of semi-automatic smoothing and segmentation. A total of 30 patients’ data were used to develop the software, and data collected from 17 patients were used for validation, of which the trajectories of 8 patients were partly masked. Pearson correlation coefficients between the manual and automatic tracking are high and statistically significant (0.942 to 0.991, *P-value*<0.0001). Relative errors between automatic tracking and manual tracking in terms of the x-axis, y-axis and 2D range of hyoid bone excursion range from 3.3% to 9.2%. We also developed an automatic method to segment each hyoid bone trajectory into four phases (elevation phase, anterior movement phase, descending phase and returning phase). The semi-automatic hyoid bone tracking from VFSS data by our software is valid compared to the conventional manual tracking method. In addition, the ability of automatic indication to switch the automatic mode to manual mode in extreme cases and calibration without attaching the radiopaque object is convenient and useful for users. Semi-automatic smoothing and segmentation provide further information for functional motion analysis which is beneficial to further statistical analysis such as functional classification and prognostication for dysphagia. Therefore, this software could provide the researchers in the field of dysphagia with a convenient, useful, and all-in-one platform for analyzing the hyoid bone motion. Further development of our method to track the other swallowing related structures or objects such as epiglottis and bolus and to carry out the 2D curve registration may be needed for a more comprehensive functional data analysis for dysphagia with big data.

## Introduction

Oropharyngeal dysphagia is caused by various diseases such as stroke, Parkinson's disease, neuromuscular diseases, and head and neck cancer[1]. The prevalence of dysphagia is expected to increase taking into consideration an ageing population and the increase of incidences of diseases related with dysphagia[2, 3].

Videofluoroscopic swallow study (VFSS) is considered to be the gold standard tool in the assessment of patients with dysphagia [4]. Most of the interpretation of VFSS in the clinical setting is qualitative or semi-quantitative and depends on subjective decision by an interpreter. Some clinicians or researchers are using temporal parameters (e.g. oral transit time, pharyngeal transit time) or kinematic parameters from motion analysis to overcome the qualitative nature of VFSS and to gain more data to classify the dysphagia, to predict the prognosis or to assess the treatment effect[5–8]. The hyoid bone is most commonly selected in kinematic analysis [9–13]. Both displacement and velocity of the hyoid bone excursion are associated with swallowing function and dysphagia; the maximum excursion and peak velocity of the hyoid bone motion are associated with bolus volume[14], the hyoid bone anterior displacement is reduced in patients with myopathy and irradiated nasopharyngeal carcinoma [5, 15] and laryngeal elevation velocity was an independent predictor of aspiration in patients with acute ischemic stroke [16]. Therefore, the parameters from hyoid bone motion analysis provide some meaningful solutions in research or clinical practices. However, the classical manual tracking methods are labour intensive and impractical in real clinical practices[17, 18].

To overcome this limitation, researchers have tried to develop a software to track the hyoid bone and to get the trajectory automatically. Recently, Patrick et al. [19] have reported their computer-assisted assessment of hyoid bone motion and found a high correlation between automatic tracking and manual tracking. This software can reduce the burdens for VFSS motion analysis and make further quantitative analysis practically possible. We developed a new algorithm and tried to resolve the limitations of the existing software. One limitation is to track the masked points. We have developed an algorithm in our software to track the masked point. Automatic smoothing and calibration have also been developed. Finally automatic segmentation of hyoid bone motion has been developed and added to the software, which is necessary for further quantitative motion analysis. The efficiency and accuracy of the automatic or semi-automatic process from hyoid bone tracking and smoothing to segmentation enables the motion analysis of VFSS to have a potential wide use in clinical practice and research. The software is validated in patients with stroke.

## Materials and methods

### Subjects and experimental design

VFSS data and medical information for stroke patients were retrospectively reviewed from the database of VFSS movie files and medical records in Seoul National University Bundang Hospital. A total of 30 patients’ data (mean age: 62.0 ± 11.4 yrs, 23 men and 7 women) were used to develop software (tracking, smoothing and calibration, and segmentation) and 20 loops from 17 patients’ trajectories (10 loops from 8 unmasked trajectories and 10 loops from 9 masked trajectories) were used to validate the semi-automatic tracking method. One loop for each subject’s trajectory of their hyoid bone was usually detected and extracted while two loops were obtained in subjects 5, 11 and 16.

VFSS was tested in subjects with dysphagia after stroke with foods in various forms, including fluid, thickened fluid, a semi-blended food and boiled rice, which was the modified protocol of Logemann[20]. Each food was fed by spoon. The lateral projection of the VFSS taken during the 2-ml thin-fluid swallowing was used for software development and validation. VFSS were recorded at frequency of 30 frames per second.

One researcher performed the manual tracking and automatic tracking of hyoid bone from VFSS clips. When one type of tracking was performed, the tester did not consult the result of another type of tracking. Another researcher, who was blind to the results obtained by the first person, performed the manual tracking again and the data were used to assess the interrater reliability. The research protocol was approved by the Seoul National University Bundang Hospital institutional review board and was conducted in accordance with the regulatory standards of Good Clinical Practice and the Declaration of Helsinki (World Medical Association Declaration of Helsinki: Ethical Principles for Medical Research Involving Human Subjects, 2000).

### Overview of the all-in-one platform for the motion analysis of hyoid bone

Inspired by the method by Kellen et al. [19], in our study we specify a target point on the hyoid bone on one frame and then track the target automatically for the whole video sequence. The ROI window size by default should be large enough so that it is not very sensitive to the smaller movements of the hyoid bone. Each frame of the sequence is then processed to track the ROI centered at the target point across frames. The tracking of the ROI, partly masked by other objects such as the mandible, in some frames has been considered in our methodology. Furthermore, we consider the situation where the tracking process might collapse due to the existence of unidentifiable and invisible ROI in some frames. An automatic monitoring and indication mechanism has been added, enabling us to re-specify the target point and reset the window size of ROI and then resume the tracking process. In order to correct for the subject’s head motion during process, a new coordinate system is defined via the anterior-interior border of the second and fourth cervical vertebrae across the entire procedure. Semi-automatic smoothing via a cubic smoothing are added for those target points in the hyoid bone and in the cervical vertebra for the sake of reducing tracking errors. Our platform also emphasizes the segmentation. After selecting one desired loop from the data, the automatic segmentation is carried out. By analyzing the first and second derivatives, a definition of splitting score is introduced and used for an automatic segmentation. This provides necessary and useful information for clinical assessment and further statistical analysis such as functional classification. The code for data tracking and for data pre-processing like semi-automatic smoothing, calibration, validation and segmentation is based on MATALAB (R2014a) and RStudio Version 0.99.484, respectively.

In summary, the main contributions of the present work include, (a) the development of a new algorithm based on the existing method by Kellen et al. [19] to track the masked part of the hyoid bone and a dynamic monitoring mechanic to fix the wrong-tracking problems in time; (b) the development of semi-automatic smoothing and calibration for reducing tracking errors; and (c) the development of a new method of automatic segmentation of hyoid bone motion, which could provide the researchers in the field of dysphagia a convenient, useful, and all-in-one platform for the analysis of hyoid bone motion.

### Procedures of tracking

To define the template ROI, the user uses the mouse to identify any target point on the hyoid bone and then one square centered at it with default side lengths can be created automatically. The target point, together with this square (called ROI or template) will be tracked automatically frame by frame, utilizing the information from horizontal and vertical edge images calculated by Sobel edge operators [21]. The key point is to minimize the sum of the squared differences between the local edge characteristics in the templates and that in the images via rotation and shift over a suitable neighborhood. The best match to the template in the next frame can then be found. The tracking process can be iterated by updating the positions of both ROI and the target point.

When it comes to extreme situations, for example the ROI or the target point on the hyoid bone being covered by other objects like the lower part of the mandible, the tracking method [19] no longer works because the local edge characteristics of the ROI are heavily interrupted. So far there has been little research on addressing this problem.

**Fig 1:**
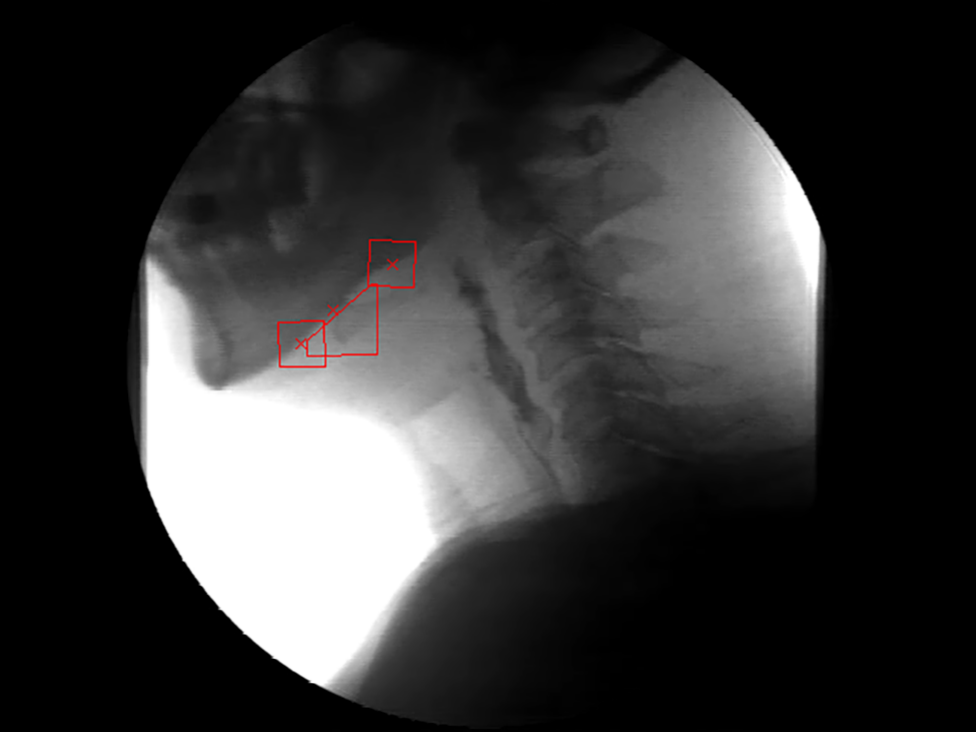
Example of tracking the partly masked ROI. The target point pinpointed by the middle red cross is covered by the mandible in this case. This ROI (the middle square) is cut off by the line segment linked by another two red crosses (their corresponding ROI’s are the upper and lower squares) along the lower edge of mandible.

Technically, the masked ROI refers to the ROI totally or partly overlapping with other objects, which often happens over just a few frames of the whole video sequences. In this case, it is hard to locate the target point even by eyes. The idea is to cut off the masked part in the current ROI. Besides the target point, it is necessary to track another two points along the edge of the lower part of the mandible. The locations of these two points should be flexible and the distance between them wide enough to guarantee that the line segment connected by them can approximate the lower edge of the mandible properly. We then check whether the current ROI crosses this line. The masked part above the line will be cut off if it crosses; otherwise, it will be treated as a normal case. A new ROI′ can therefore be obtained using the normal tracking procedure (Fig 1). Another key point is the requirement of averaging the template matching error over the number of pixels within the new ROI′.

Assuming *i*_0_ is the number of the reference frame, in which the template ROI(*i*_0_) is produced, and *i* represents the current frame number, the template in the current frame is supposed to move with a rotation angle $\tilde{\theta }$ and shift ${\left(\tilde{x},\tilde{y}\right)}^{T}$. After this transformation, the removal of the hidden part of the template (Fig 1) is required. As a result, a new ROI′(*i* + 1) in the (i+1)th frame, which is not a square any more, is obtained. All the x coordinates and the corresponding y coordinates of the pixels within ROI′(*i* + 1) are saved in the vectors ROI′_**x**_(i + 1) and ROI′_**y**_(i + 1) respectively. The ROI(*i*_0_) needs to be changed to ROI′(*i*_0_) in the same way to make their local edge characteristics *E*_*x*_ and *E*_*y*_ comparable. The template matching error $\Delta (\tilde{x},\tilde{y};\tilde{\theta })$ is then given by:

Δ(x˜,y˜;θ˜)=1N(i+1)∑x∈ROI′x(i0);y∈ROI′y(i0)x′∈ROI′x(i+1);y′∈ROI′y(i+1)(Ex(x,y,i0)−Ex(x′,y′,i+1))2+(Ey(x,y,i0)−Ey(x′,y′,i+1))2

where *N*(*i* + 1) represents the total number of the pixels within ROI′(*i* + 1). This equation is minimized by a global optimization method GA with a constrained search space [22]. In our implementation, the search constraints are: -2.5*π*/180≤$\tilde{\theta }$≤2.5*π*/180, and $\text{-}5\leq \tilde{x},,\tilde{y}\leq 5$. The tracking for the next position of the template and target point in this special case can then progress. However, the tracking process may become unstable if the ROI is totally masked by the mandible. In this rare case, we may estimate the underlying point by the points tracked in nearby frames or to track it manually.

**Fig 2:**
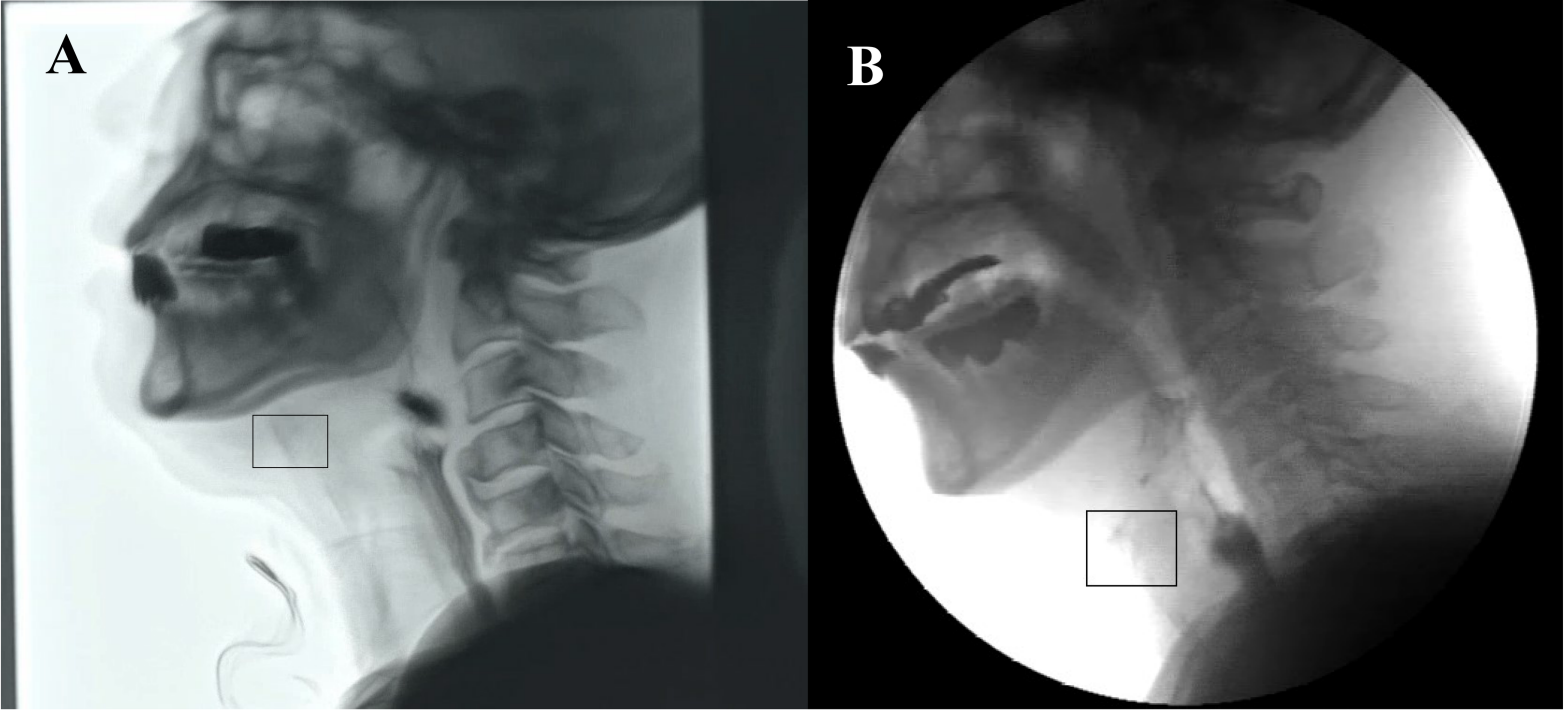
Example of unrecognizable hyoid bone located in the square in black. A. The hyoid bone moves too fast, resulting in the almost equal grey scale value in the area around it. B. The strong reflective light makes the hyoid bone invisible.

In some rare situations, such as the hyoid bone moving suddenly or too fast, the target point is hardly recognizable (Fig 2). The optimal search is not applicable to those circumstances; therefore, a sensitive monitoring mechanic should be used to avoid possible wrong tracking. Kellen et al. [19] used a prediction model to initialize the new point position for the sake of improving tracking accuracy. We used a similar idea but for monitoring purpose in our package. The prediction model and displacement error for the next position of the target point are given by:

$$\tilde{H}(i+1)\approx H(i)+\dot{H}(i)\Delta t+\frac{\ddot{H}(i)}{2}{\left(\Delta t\right)}^{2}+\frac{\dddot{H}(i)}{6}{\left(\Delta t\right)}^{3}$$

and

$$\Delta =|\tilde{H}(i+1)-H(i+1)|$$

where Δ*t* is the inter frame period, *i* is the current frame number, *H*(*i*) and *H*(*i* + 1) are the hyoid bone’s current and next position, and $\dot{H}(i)$, $\ddot{H}(i)$ and $\dddot{H}(i)$ are respectively the first, second and third derivative of *H*(*i*). Given the previously acquired *H*(*i*), the Δ (absolute difference between $\tilde{H}(i+1)$ and the current *H*(*i* + 1)) should be less than a threshold (8 pixel units in our implementation). Otherwise, the tracking will be regarded as a failure. The software will automatically review the possible wrong-tracking frames backwards and forwards to identify the accurate sequence number of the first failure frame. After confirming it, we can manually adjust the window size of *ROI*(*i*), and go back to the tracking procedure by re-specifying the same target point in the *i*th frame by mouse click and continue the tracking process.

### Smoothing and calibration

**Fig 3:**
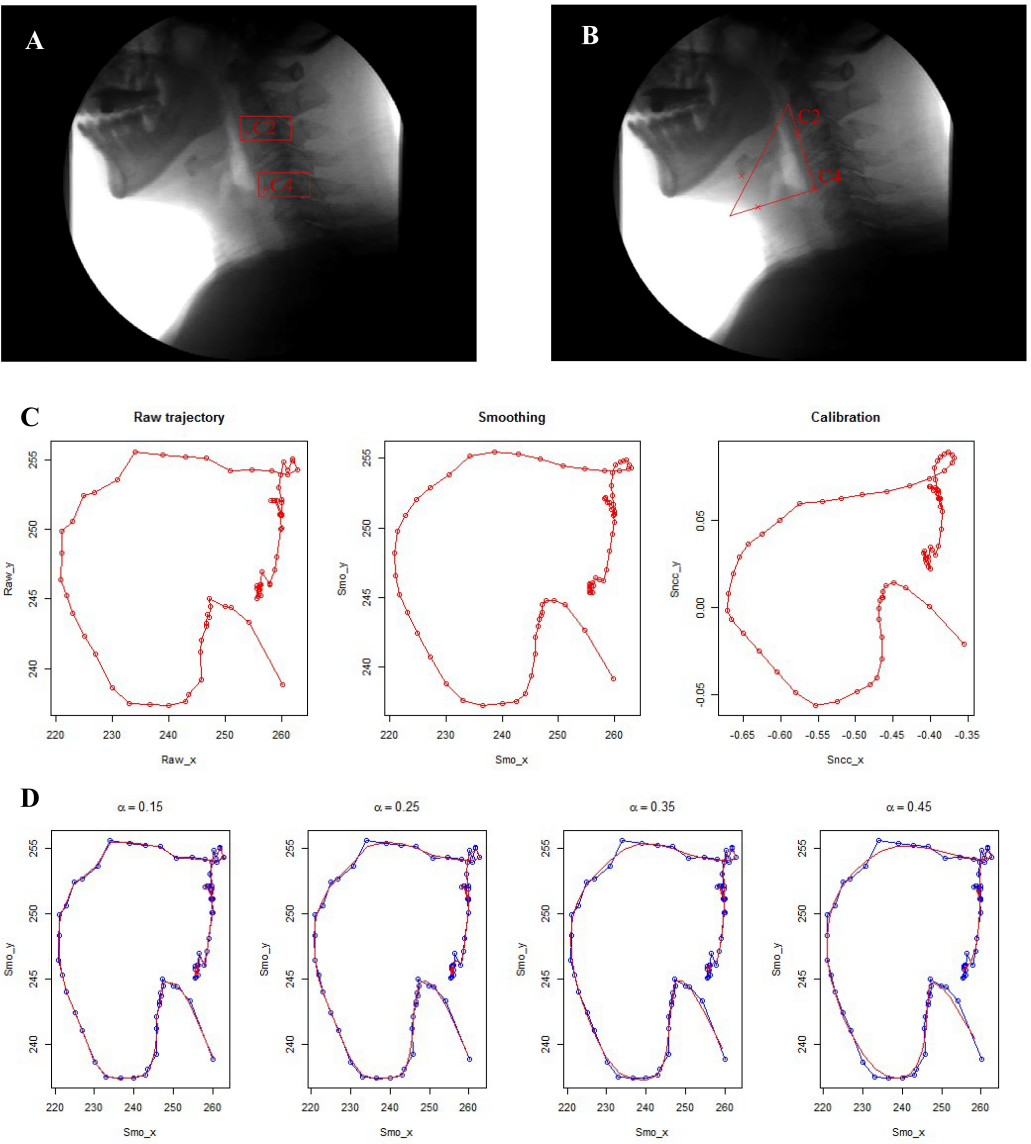
Example of smoothing and calibration. A. Manually specified two points, C2 and C4, indicated as two red crosses, in the anterior-interior border of the second and the fourth cervical vertebra at the beginning of tracking. B. The patient-centric coordinate system with the origin C4, where the y axis is defined as the line crossing C4 and C2 upward and the x axis is defined as the line vertical to the y axis leftward. C. The rugged trajectory in the left panel is raw data based on the image-centric coordinate system (in pixels) while the smoothing one in the middle and the calibrated one in the right panel which is based on a patient-centric coordinate system (in CU). D. Semi-automatic smoothing by adjusting the spline parameter, which ranges from 0.15 to 0.45. Blue curves represent the raw trajectory while the red ones are smoothing curves.

The tracking described in the previous subsections is based on the coordinate system of which the origin is located at the bottom-left corner of the image (image-based coordinate system). The problem is that there is usually a huge body or head motion which will blur the movement of hyoid bone during the swallowing process. Removal of this kind of error can be done by using a new patient-centric coordinate system [19, 23]. Practically, it seems to be much more convenient and more efficient to define the new coordinate system that is just based on two special points defined in the following way. As described by the previous study [24], the y-axis of the patient-centric coordinate system is defined as a straight line connecting the anterior-interior border of the fourth cervical vertebra to that of the second cervical vertebra. The x-axis is defined as a line vertical to the y-axis crossing the origin, C4, as seen in Fig 3B. The points C4$\left(x_{c_{4}},y_{c_{4}}\right)$ and C2$\left(x_{c_{2}},y_{c_{2}}\right)$ can be tracked at the same time using the methods illustrated in the previous subsections over the entire video sequences (Fig 3A). To reduce the tracking errors, smoothing is carried out for both the target point in the hyoid bone and the two tracking points in the cervical vertebra using a cubic smoothing spline. The degree of smoothing is controlled by the smoothing parameter, which can be adjusted by the operator to avoid over-fitting (Fig 3D). Then all the data is calibrated by the vertical distance from C4 to C2. Given those two points, the target point *H*(*x*,*y*) in the image-based coordinate system can be transformed to *H*′(*x*′,*y*′) in the patient-centric coordinate system by a simple rotation and shift. The formula is given by:

$$\left(\begin{array}{c} x\prime \\ y\prime \end{array}\right)=\frac{1}{\left|C2-C4\right|}\left(\begin{array}{cc} cos(\theta ) & sin(\theta )\\ -sin(\theta )) & cos(\theta ) \end{array}\right)\left(\begin{array}{c} x-x_{c_{4}}\\ y-y_{c_{4}} \end{array}\right)$$

where

$$\theta =\frac{\pi }{2}+arctan\left(\frac{x_{c_{4}}-x_{c_{2}}}{y_{c_{4}}-y_{c_{2}}}\right),\;|C2-C4|=\sqrt{{\left(x_{c_{4}}-x_{c_{2}}\right)}^{2}+{\left(y_{c_{4}}-y_{c_{2}}\right)}^{2}}.$$

The effect of smoothing on reducing tracking errors is demonstrated in Fig 3C. The calibration procedure aims to reduce the errors caused by head motion and make the data collected from different subjects comparable. Our later data analysis will be based on the trajectory after smoothing and calibration, of which the coordinate is based on cervical units (CU) (One cervical unit is defined as the distance (in pixel) between C2 and C4, i.e. |*C*2−*C*4|).

### Segmentation

Dividing one loop of the trajectory into certain phases is necessary for the assessment of the hyoid bone movement and useful for further statistical analysis. However, little research has been carried out in this area, particularly on automatic segmentation. In terms of the concept of phases, Isao Kaneko [22] performs a quantitative study dividing manually the movement into 5 phases:1^st^ elevation phase, 2^nd^ elevation phase, static phase and 1^st^ and 2^nd^ descending phase. Koichi Yabunaka et al. [25] have also done similar work on sonographic assessment of the hyoid bone movement during swallowing by segmenting the movement into 4 phases: Elevation, Anterior, Remain and Return. We developed a semi-automatic segmentation method. After manually selecting one complete desired loop from the entire raw trajectory, an automatic segmentation is conducted.

**Fig 4:**
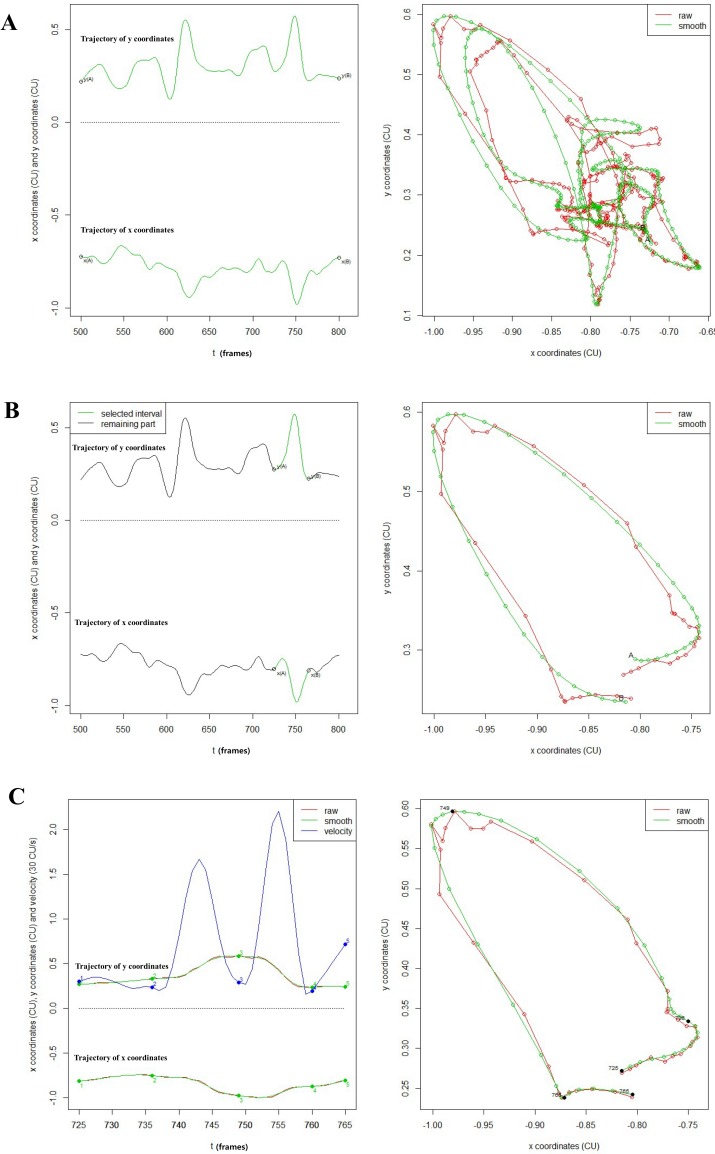
Example of automatic segmentation for one non-aspiration case. A. The curves of the x coordinates and y coordinates of all the data (the left panel) and the entire 2D trajectory (the right panel): the dots connected with a line in red show the raw trajectory just after being calibrated while the ones in green represent smoothing data after calibration. B. Extracted one loop based on the two cutting points A and B (the left panel) from the entire trajectory and the corresponding 2D trajectory(the right panel). C. Automatically segmenting the trajectory into four phases. The upper curve in green in the left panel stands for the smoothing y coordinates and the lower one for the smoothing x coordinates, together with the curves in red representing raw data. The curve in blue represents the velocity amplitude *v*(*t*), where t represents the video frame sequence and the numbers in different colours stand for splitting points’ order. The right panel shows the segmentation results in 2D trajectory.

For simplicity, we use points (*x*(*t*),*y*(*t*)) to represent x and y coordinates after calibration and smoothing in the *t*th frame. Two ends of the manually identified complete loop are denoted by *t*_*A*_ and *t*_*B*_. The technique is to acquire one desired time interval including one peak in *y*(*t*) and one valley in *x*(*t*) at the same time. These two points can be easily chosen by human eyes. For instance, the left panel of Fig 4A shows that it is easy to identify the peaks and valleys in these two marginal curves. The end points *t*_*A*_ and *t*_*B*_ are chosen such that the interval (*t*_*A*_,*t*_*B*_) contains both one peak in the upper curve (*y*(*t*)) and one valley in the lower curve (*x*(*t*)). Furthermore, the distance between (*x*(*t*_*A*_),*y*(*t*_*A*_)) and (*x*(*t*_*B*_),*y*(*t*_*B*_)) should be as small as possible (see left panel of Fig 4B). The ideal situation is that the distance equals to zero, i.e., the starting point *A* and ending point *B* overlap.

Once the desired loop is obtained, automatically dividing the movement into different phases over (*t*_*A*_,*t*_*B*_) can be carried out via analyzing the corresponding velocity amplitude $v(t)=\sqrt{{\left(\frac{dx(t)}{dt}\right)}^{2}+{\left(\frac{dx(t)}{dt}\right)}^{2}}$, *t* ∈ (*t*_*A*_,*t*_*B*_). Specifically we can find the splitting points t from the following equations:

$$(A)\;\frac{dv(t)}{dt}=0;$$

$$(B)\;\frac{d^{2}v(t)}{{dt}^{2}}>0.$$

Conditions (A) and (B) can guarantee in finding all the local minima in the velocity curve (see the curve in blue in the left panel of Fig 4C). Those local minima are interesting splitting points, which can be directly used in segmentation in most cases. The corresponding segmentation result is shown in the right panel of Fig 4C. In this case the three splitting points, as well as the start point and end point, are used to split the trajectory into four phases: elevation phase, anterior movement phase, descending phase and returning phase.

**Fig 5:**
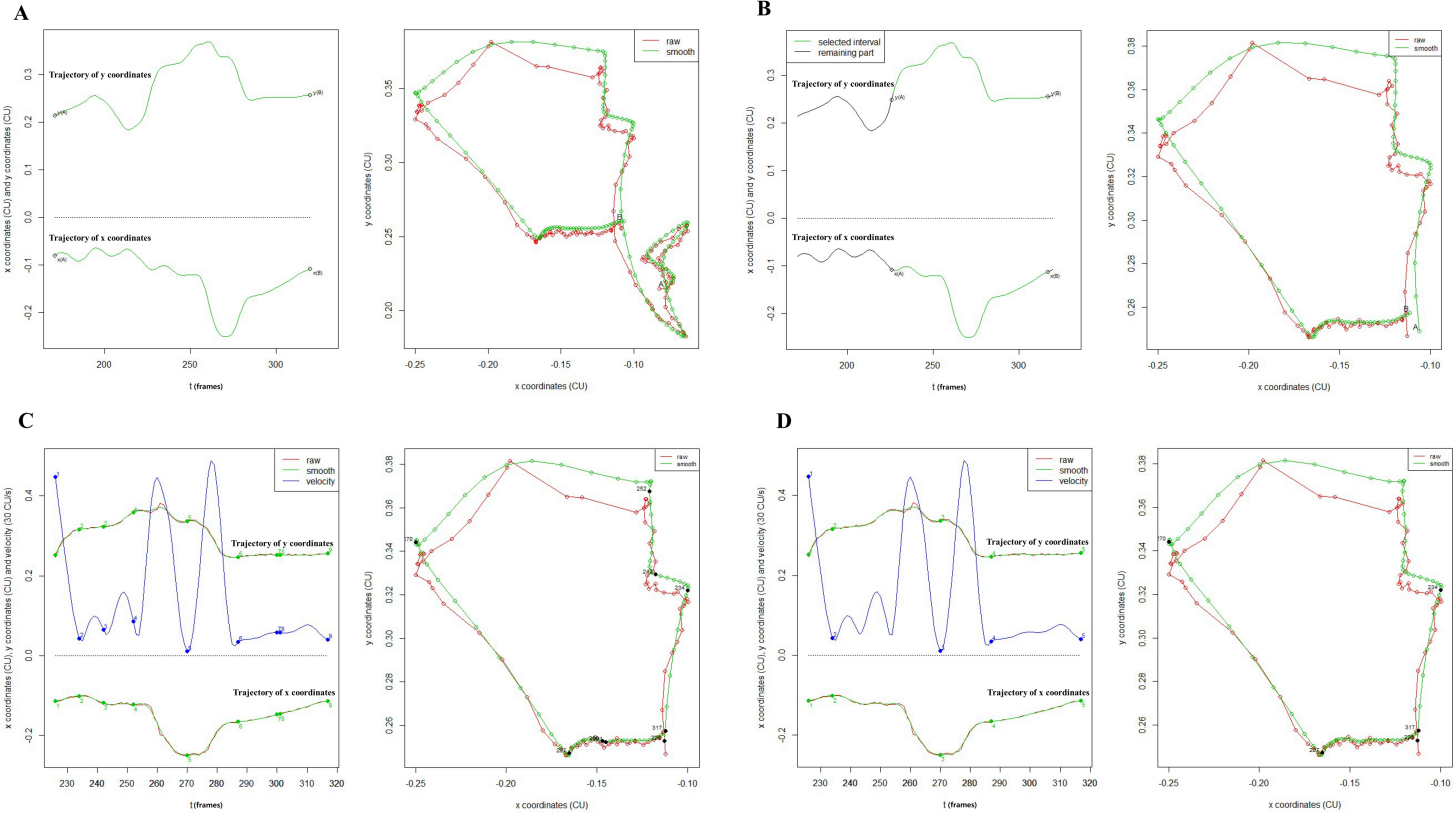
Example of automatic segmentation. A. All the data and the entire trajectory. B. Extracting one loop from the entire trajectory. C. Automatically splitting the trajectory via choosing points satisfying the condition A and B. Those numbers 2, 3, 4, 5, 6, 7, 8 on the curves in the left panel are the candidate splitting point corresponding to the points in black on the 2D trajectory in the right panel. D. Further automatically segmenting the trajectory into four phases via selecting three splitting points from C. The selected points 2, 3, 4 in the left panel of D are equivalent to the points 2, 5, 6 in C.

It is not uncommon that more than three candidate splitting points could be obtained based on the conditions (A) and (B) (Fig 5C), particularly for stroke patients. Therefore, we propose to identify the three best splitting points based on a new measurement, namely, splitting score. Assume there exist *n*−2 candidate splitting points: *t*_2_,*t*_3_,⋯*t*_*n*−1_ (in addition of the start point *t*_1_ and end point *t*_*n*_). For the *i*th splitting point *t*_*i*_ (*i* ∈ [2,*n*−1]), using the velocity amplitude *v*(*t*_*i*_), we can define the following measures.

(a) Forward Splitting Score:

$$FSS(t_{i})=\max\left(v(t)\right)-v(t_{i}),t\in [t_{i-1},t_{i}]$$

(b) Backward Splitting Score:

$$BSS(t_{i})=\max\left(v(t)\right)-v(t_{i}),t\in [t_{i},t_{i+1}]$$

(c) Splitting Score:

$$SS(t_{i})=FSS(t_{i})+BSS(t_{i})$$

Actually, the *SS* value can be regarded as the turning intensity for the candidate points, the larger, the better. Those *t*_*i*_’s with the top 3 Splitting Scores are chosen as the desired splitting points. Fig 5C shows that there are 6 candidate splitting points satisfying the conditions of (A) and (B). It is easy to identify the three points, indicated as label 2, 5, 6, based on the top 3 SS values. The result is shown in Fig 5D.

### Validation and statistical analyses

We tracked 20 loops from 17 subjects using our semi-automatic tracking methodology. Next, each swallow was also tracked manually by two independent trained observers, who were instructed to track one recognizable and fixed target point on the hyoid bone across all frames by clicking mouse. For the same swallow, we compare the two different trajectories tracked by automatic computer-assisted method and manually by one of those two observers. Similar to the previous study [19], we used Pearson correlation coefficients, as well as the range of motion (ROM) in the x-axis direction, y-axis direction and the whole 2D situation, to measure the difference. Relative errors (%) were used to measure the degree of agreement: $\frac{\left|ROM\;in\;automatic\;tracking–ROM\;in\;manual\;tracking\right|}{ROM\;in\;manual\;tracking}$ × 100%. Apart from the raw trajectories (raw data without smoothing and calibration), five more comparisons were considered in our validation: RawNC (Raw data transforming to a new coordinate system without being scaled), RawNCC (Raw data with both coordinate system alternation and scaling), Smo (Raw data with smoothing), SmoNC (RawNC data with smoothing), SmoNCC (RawNCC data with smoothing). Continuous variables are presented as mean ± 1 SD. Parameters generated were compared between aspiration and non-aspiration groups using an independent t-test. Furthermore, in order to justify the current validation methodology, the Intraclass correlation and Pearson correlation are utilized to measure the inter-rater reliability between those two observers. We calculate those two measurements for both x coordinates and y coordinates of three points’ locations in each frame by two raters’ manual tracking. As mentioned before, those points are respectively located at the bottom left of the hyoid bone, anterior-interior border of the second cervical vertebra and that of the fourth cervical vertebra. Statistical analysis was performed using RStudio Version 0.99.484. Raw data used for the analysis are available from S2, S3 and S4 Files.

## Results

**Fig 6:**
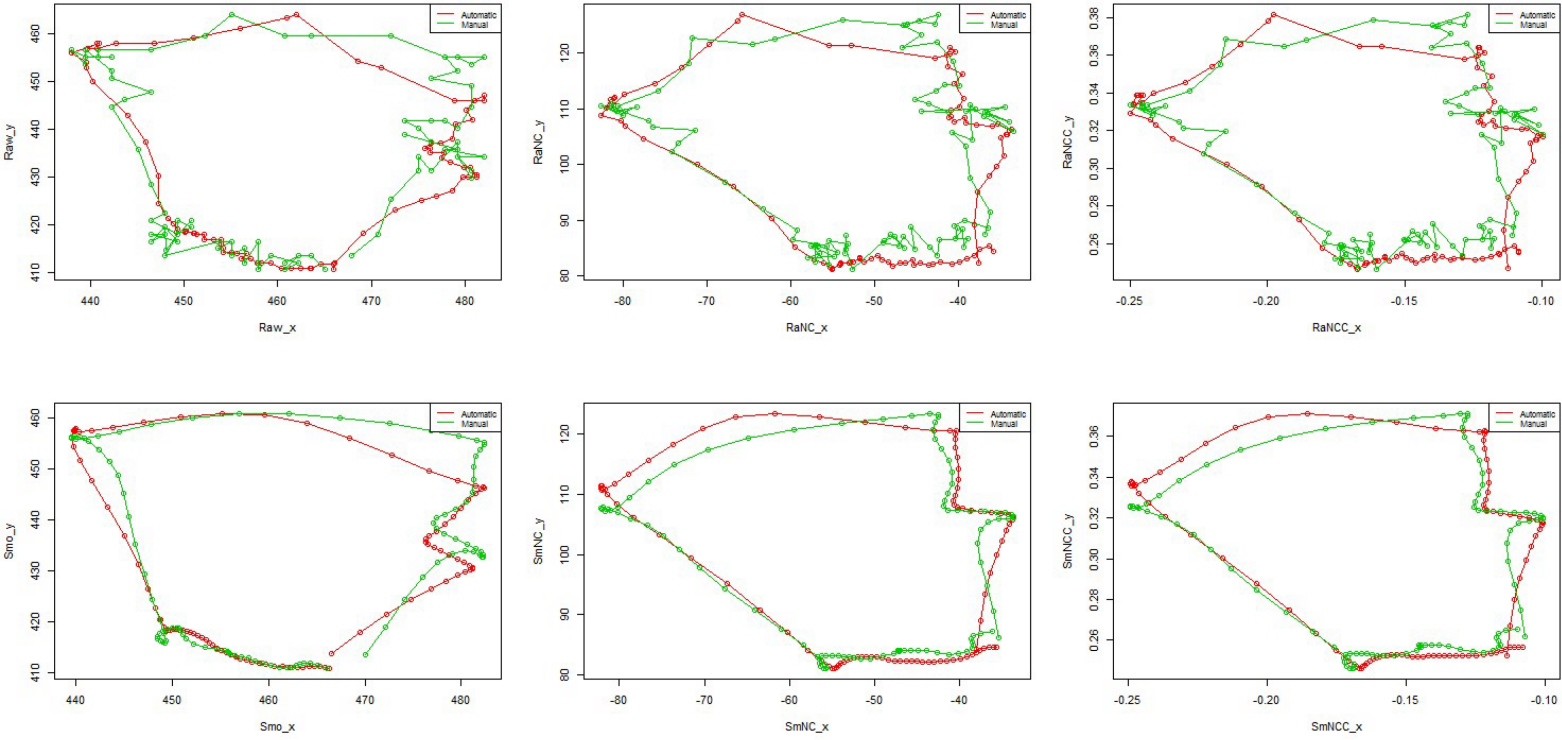
One example from the unmasked group. The hyoid bone trajectories in red are based on semi-automatic tracking methodology while those in green are by manual method.

**Fig 7:**
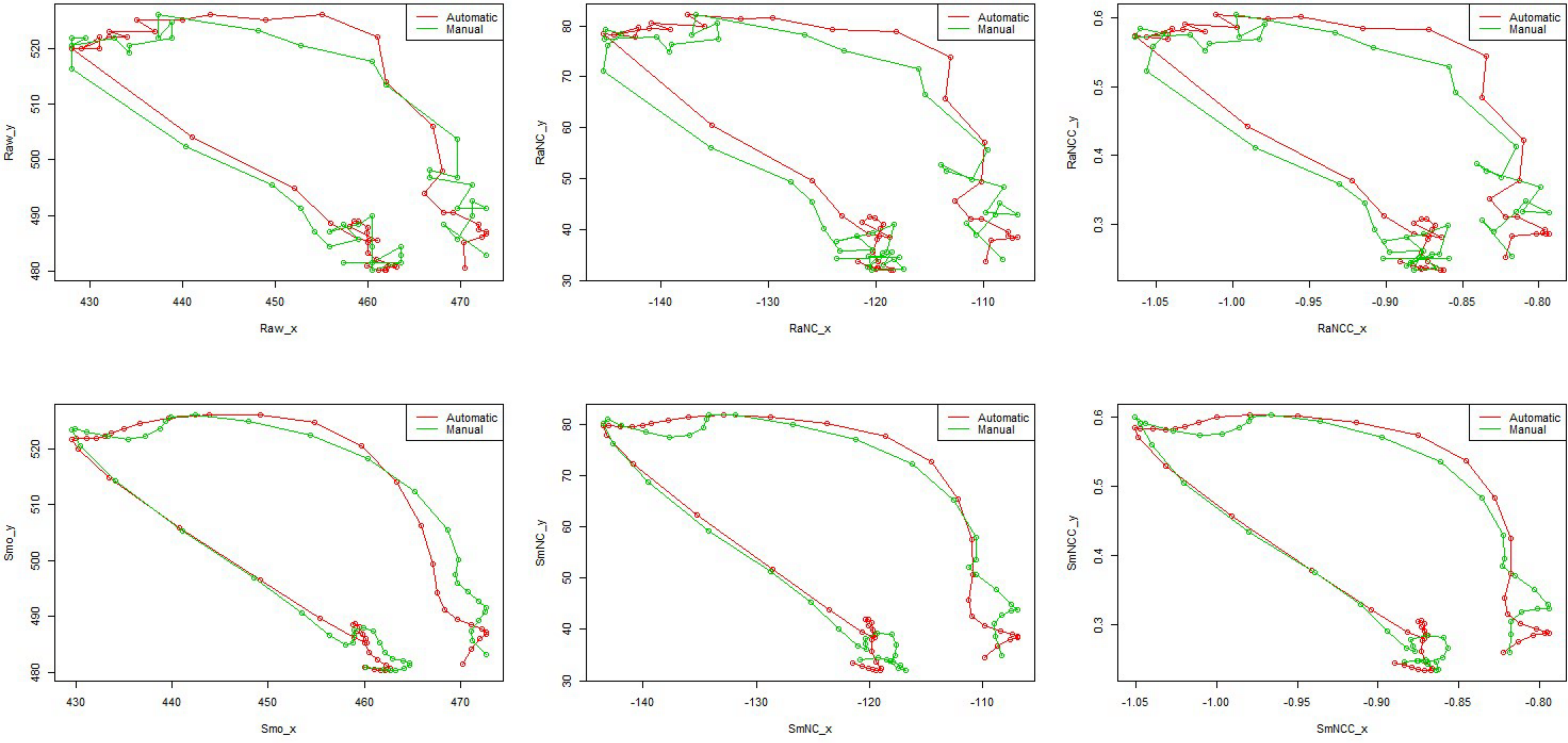
One example from the masked group. The hyoid bone trajectories in red are based on semi-automatic tracking methodology while those in green are by manual method.

Fig 6 and Fig 7 show both computer defined and manual defined trajectories corresponding to six cases for two typical data sets, one from the unmasked group and the other from the masked group. Overall, two trajectories in each case match well (high Pearson correlation coefficients and low relative errors between manual tracking and automatic tracking in Table 1). The slight difference may be caused by different target points on the hyoid bone identified by the computer and the trained observer.

**Table 1:** Pearson correlation coefficients between two methods and relative errors (%) from two methods.

Cases	Pearson r (x-axis)	Pearson r (y-axis)	ROM in automatic tracking (x-axis)	ROM in manual tracking (x-axis)	Relative errors % (x-axis)	ROM in automatic tracking (y-axis)	ROM in manual tracking (y-axis)	Relative errors % (y-axis)	ROM in automatic tracking (2D)	ROM in manual tracking (2D)	Relative errors % (2D)
**Unmasked group (10** loops **from 8 trajectories)**											
Raw	0.982	0.982	55.22±15.06	57.57±13.87	6.1±4.7	53.96±26.69	55.77±26.49	6.5±4.7	70.72±23.31	72.67±23.45	5.6±4.1
RawNC	0.977	0.954	54.98±16.21	56.87±14.58	8.7±5.0	55.06±27.24	56.39±25.27	6.5±4.5	71.50±23.65	72.54±22.45	5.9±3.9
RawNCC	0.977	0.958	0.27±0.10	0.29±0.11	9.2±6.6	0.27±0.18	0.29±0.18	8.6±6.0	0.36±0.17	0.38±0.18	6.9±4.8
Smo	0.991	0.990	55.01±15.01	55.00±14.63	4.8±3.8	53.16±26.53	53.12±26.55	4.6±3.1	70.53±22.80	69.88±23.62	4.6±3.4
SmoNC	0.984	0.967	54.49±16.19	53.58±14.41	6.5±4.6	53.95±26.96	53.67±25.97	4.8±4.6	71.35±23.33	69.90±23.12	4.8±3.9
SmoNCC	0.984	0.971	0.27±0.10	0.28±0.11	7.6±5.4	0.27±0.17	0.28±0.18	5.4±4.6	0.36±0.17	0.37±0.18	5.4±3.3
**Masked group (10 loops from 9 trajectories)**											
Raw	0.975	0.965	56.23±22.04	58.44±22.34	6.1±5.1	41.80±16.06	43.01±15.89	5.0±4.3	63.80±22.52	65.89±23.32	5.3±5.8
RawNC	0.972	0.946	56.00±18.39	57.30±16.89	7.3±6.5	41.63±15.46	43.48±15.32	8.5±7.8	64.01±21.30	64.24±18.38	6.4±5.4
RawNCC	0.969	0.942	0.31±0.13	0.33±0.14	8.6±7.6	0.22±0.10	0.23±0.11	7.1±6.5	0.356±0.14	0.36±0.14	6.0±6.2
Smo	0.982	0.978	55.50±22.41	56.11±22.00	4.3±4.0	40.66±16.43	41.03±15.51	3.3±4.1	62.68±22.46	63.53±22.73	4.1±4.7
SmoNC	0.979	0.961	55.14±18.49	55.37±17.23	5.0±5.6	40.35±15.89	41.67±15.03	6.9±7.1	63.12±21.44	62.56±18.48	6.2±4.3
SmoNCC	0.975	0.957	0.31±0.13	0.32±0.14	6.8±6.9	0.21±0.10	0.22±0.10	5.8±6.0	0.35±0.15	0.35±0.14	5.5±4.9

2D-2 dimension, ROM-range of motion, Raw-raw data without smoothing and calibration, RawNC-raw data transformed to a new coordinate system yet without being scaled, RawNCC-raw data with complete calibration, Smo-raw data with smoothing, SmoNC-RawNC data with smoothing, SmoNCC-RawNCC data with smoothing. Values are Pearson correlation coefficients or mean ± SD. *P*-values for all Pearson correlation coefficient were less than 0.0001.

Table 1 shows Pearson correlation coefficients and relative errors between manual tracking and automatic tracking, and range of motion from each tracking method. We can see that all of coefficients are in the interval between 0.942 and 0.991 (*P-value*<0.0001) and the relative errors in terms of x-axis, y-axis and 2D range of hyoid bone excursion range from 3.3% to 9.2%. Overall, the case of proper smoothing usually outperforms the other.

Table 2 shows the average of Intraclass correlation and Pearson correlation coefficients for both x coordinates and y coordinates range from 0.995 to 0.999 (*P-value*<0.0001). It indicates a high consistency of quantitative measurements made by those two independent trained observers, which provides a justification of the methodological errors in our study.

**Table 2:** Average Pearson correlation coefficients and Intraclass correlation coefficient (ICC) between two independent observers for measuring the inter-rater reliability.

Tracking results	Methods	Estimate	95% CI	*P* value
X coordinates	Pearson’s r	0.999	(0.998, 0.999)	<0.0001
	ICC	0.998	(0.998, 0.999)	<0.0001
Y coordinates	Pearson’s r	0.998	(0.998, 0.998)	<0.0001
	ICC	0.996	(0.995, 0.996)	<0.0001

**Fig 8:**
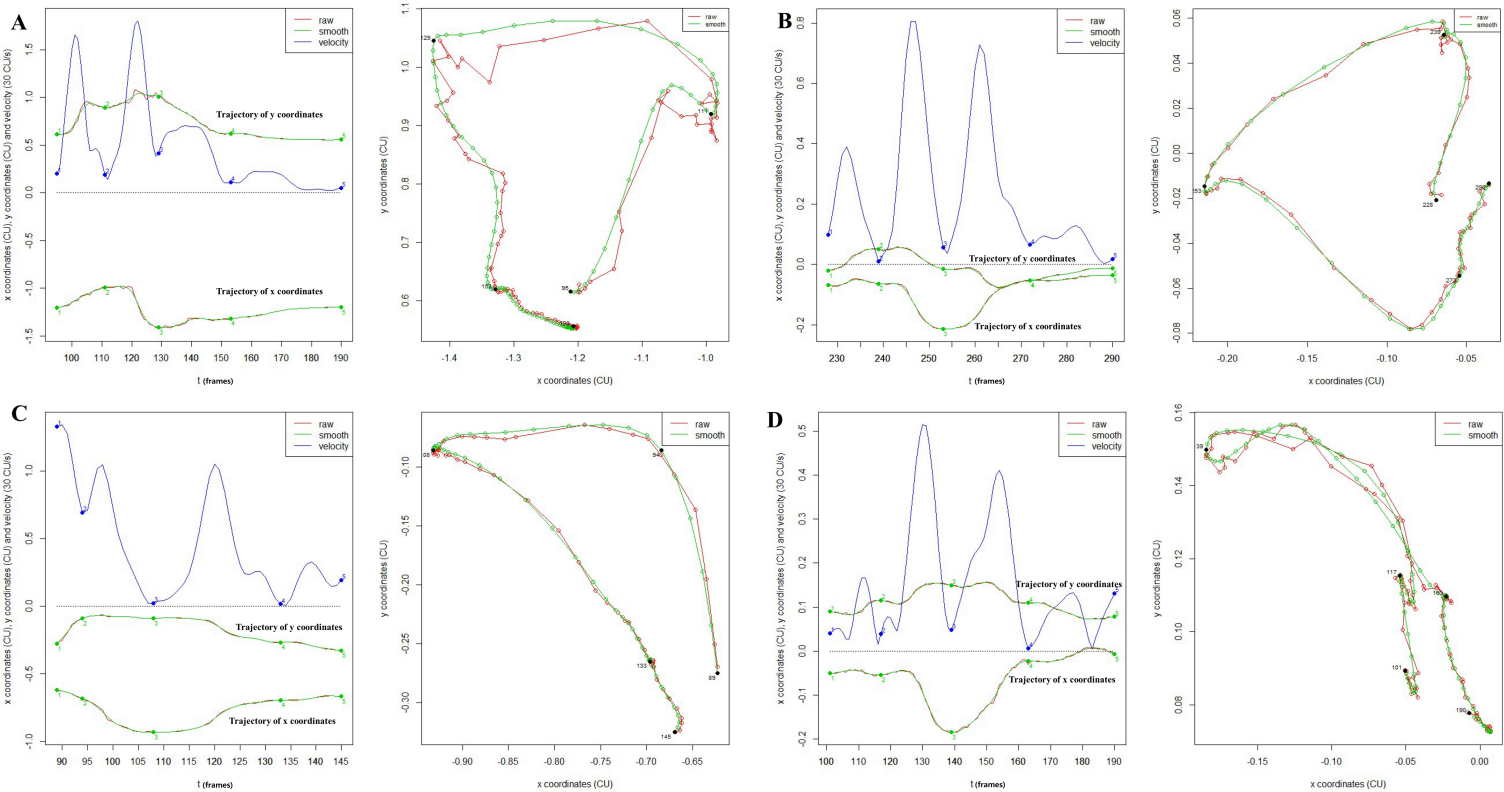
Examples of segmentation. A. Successful automatic segmentation to four phases. B. Failed automatic segmentation but successful manual segmentation to four phases. C. Manual segmentation to 3 phases (no returning phase). D. Failed segmentation due to abnormal trajectory.

According to our automatic segmentation method, most of the hyoid bone motion (14 out of 30 subjects) can be segmented automatically into four phases (Fig 8A and S1 File). For subjects 5, 7, 8, 22, 23 and 24, there is an error in automatic segmentation but the four phases can be segmented manually (Fig 8B and S1 File). For subjects 3, 9, 14 and 18, only 3 phases can be segmented (no returning phase) (Fig 8C and S1 File). The trajectories of the remaining six subjects (number 4, 15, 20, 26, 29, 31) are very abnormal and cannot be segmented to any typical phases (Fig 8D and S1 File).

## Discussion

In this study with VFSS data from stroke patients, the trajectories tracked by our semiautomatic tracking method show high agreements (high Pearson correlation coefficient and low relative errors) with those obtained by the manual tracking (Table 1). In addition, the new automatic algorithm used to track the masked target points by the mandible also shows high agreement with the manual tracking (Fig 7 and Table 1).

Our software has several potential advantages in tracking the hyoid bone, compared to the previous ones. First, the marker to track the hyoid bone [26] is not applied in this method. The marker attachment is uncomfortable for a patient, and different marker positions and detachments during swallowing might result in biased measurements. Second, we use the cervical spine height as the reference distance unit for calibration and demonstrate that this method works reliably in practice. The number of pixels used as the distance in the previous study [19], cannot represent the real distance because the real distance per one pixel can vary depending on the angle and distance from the x-ray source to the subject. Some studies use radiopaque markers such as coins [5] and it can more precisely calculate the exact distance. However, the marker attachment is not comfortable and perpendicular x-ray projection has to be warranted. In addition, it is recently recommended to use the normalized units (e.g. percentage of the distance between C2 and C4 vertebrae), in order to reduce the magnification artifacts or sex-based differences [10, 27]. Therefore, the calibration method used in our software may be more suitable for further statistical analysis with large data. Third, the ability to track the masked point can save a lot of since no manual interfering is required during the automatic tracking. Fourth, our software can detect and trigger the automatic tracking failure and provides a chance to fix the problem manually. For example, in rare cases such as those shown in Fig 2, automatic tracking is not possible. Our algorithm can automatically notify the frame with error enabling save a lot of researcher’s time. Fifth, we add the semi-automatic smoothing process. The software aims at capturing the target points separately in each frame from the VFSS data, resulting in the rough trajectory connected by those points. In order to reduce the measurable errors, a semi-automatic smoothing algorithm was applied. The automatically smoothed wave can be reviewed and adjusted by the operator. Overall, this semiautomatic algorithm provides a way to track the motion more precisely, conveniently and efficiently.

The motion of anatomical structures such as the hyoid bone and epiglottis are related with the severity of dysphagia or treatment effect [28, 29]. Motion data from VFSS can be used for further studies using complex statistical methods, e.g. functional classification with machine learning or a prognostic model. Segmentation and registration are the further required process for the functional data analyses [30]. For instance, quantitative neuroimaging research often requires the segmentation of brain imaging data [31]. However, the manual segmentation is too time-consuming and is unreliable [32]. In the dysphagia research, segmentation process is applied in the data from accelerometer or acoustic signal [33, 34] but no study has used the segmentation process in the VFSS motion 2D data. From one loop of hyoid bone motion trajectory and velocity curve, four phases can be divided automatically (Fig 5 and S1 File) using our software. Tracking various types of hyoid bone trajectories during swallowing in dysphagia patients and obtaining the characteristics and features after the segmentation process can help researchers for further study.

Our software needs to be further developed to improve the calibration process. Except the body and head motion, Emsudina et al. [32] also consider the effect of the position and the movement of the lower edge of the mandible on the hyoid bone motion. Those methods may improve the ability to accurately track the motion of an anatomic structure in the head and neck, but it requires a much more complicated system including more fixed points’ position and particular directions in each frame while doing the calibration. It is not our main task of the current study but will be considered in our future work.

We also need to carry out 2D curve registration for the hyoid bone motion data. The deformations or displacements, termed phase variation, always arise in those curves, which can be shown through the different locations of splitting points while doing automatic segmentation (S1 File). The presence of phase variability often increases data variance and alters underlying data structures [35], we will use landmark registration or other methods, such as similarity index based registration method [36], SRV and FR Metric based multi-dimensional alignment method [37]. The tracking of other structures such as epiglottis and radiopaque bolus is also on ours. Those further developments can automatically make the output ready for further study using complex statistical methods. Using cloud computing systems, such as Microsoft Azure ML, Amazon Machine Learning or IBM Watson Analytics, can facilitate developing the dysphagia functional classification system or prognostic model with VFSS motion and clinical data, to assess new patients whenever the data is uploaded.

## Supporting information

S1 File Validation and automatic segmentation results of hyoid bone tracking for each subject (n = 30). (DOCX)

S2 File Raw data from the semi-automatic tracking used for analysis. (ZIP)

S3 File Raw data from the manual tracking used for analysis. (ZIP)

S4 File Raw data used for analyzing the interrater reliability in the manual tracking. (ZIP)

## References

[1] Abdel Jalil AA, Katzka DA, Castell DO. Approach to the patient with dysphagia. Am J Med. 2015;128(10):1138 e17-23. 10.1016/j.amjmed.2015.04.026 26007674

[2] Feigin VL, Lawes CM, Bennett DA, Anderson CS. Stroke epidemiology: a review of population-based studies of incidence, prevalence, and case-fatality in the late 20th century. Lancet Neurol. 2003;2(1):43–53. 12849300 10.1016/s1474-4422(03)00266-7

[3] Dorsey ER, Constantinescu R, Thompson JP, Biglan KM, Holloway RG, Kieburtz K, et al Projected number of people with Parkinson disease in the most populous nations, 2005 through 2030. Neurology. 2007;68(5):384–6. doi: 10.1212/01.wnl.0000247740.47667.03 17082464 10.1212/01.wnl.0000247740.47667.03

[4] Belafsky PC, Kuhn MA. The Videofluoroscopic Swallow Study Technique and Protocol. The Clinician's Guide to Swallowing Fluoroscopy: Springer; 2014 p. 7–13.

[5] Paik NJ, Kim SJ, Lee HJ, Jeon JY, Lim JY, Han TR. Movement of the hyoid bone and the epiglottis during swallowing in patients with dysphagia from different etiologies. J Electromyogr Kinesiol. 2008;18(2):329–35. doi: 10.1016/j.jelekin.2006.09.011 17187991 10.1016/j.jelekin.2006.09.011

[6] Nam HS, Beom J, Oh BM, Han TR. Kinematic effects of hyolaryngeal electrical stimulation therapy on hyoid excursion and laryngeal elevation. Dysphagia. 2013;28(4):548–56. doi: 10.1007/s00455-013-9465-x 23605128 10.1007/s00455-013-9465-x

[7] Seo HG, Oh BM, Han TR. Longitudinal changes of the swallowing process in subacute stroke patients with aspiration. Dysphagia. 2011;26(1):41–8. doi: 10.1007/s00455-009-9265-5 20058029 10.1007/s00455-009-9265-5

[8] Molfenter SM, Steele CM. Kinematic and temporal factors associated with penetration-aspiration in swallowing liquids. Dysphagia. 2014;29(2):269–76. doi: 10.1007/s00455-013-9506-5 24445381 10.1007/s00455-013-9506-5 PMC4315312

[9] Humbert IA, Lokhande A, Christopherson H, German R, Stone A. Adaptation of swallowing hyo-laryngeal kinematics is distinct in oral vs. pharyngeal sensory processing. J Appl Physiol. 2012;112(10):1698–705. doi: 10.1152/japplphysiol.01534.2011 22403349 10.1152/japplphysiol.01534.2011 PMC3365865

[10] Kraaijenga SA, van der Molen L, Heemsbergen WD, Remmerswaal GB, Hilgers FJ, van den Brekel MW. Hyoid bone displacement as parameter for swallowing impairment in patients treated for advanced head and neck cancer. Eur Arch Otorhinolaryngol. 2017;274(2):597–606. doi: 10.1007/s00405-016-4029-y 27086361 10.1007/s00405-016-4029-y

[11] Molfenter SM, Steele CM. Kinematic and temporal factors associated with penetration–aspiration in swallowing liquids. Dysphagia. 2014;29(2):269–76. doi: 10.1007/s00455-013-9506-5 24445381 10.1007/s00455-013-9506-5 PMC4315312

[12] Seo HG, Kim J-G, Nam HS, Lee WH, Han TR, Oh B-M. Swallowing Function and Kinematics in Stroke Patients with Tracheostomies. Dysphagia. 2017;32(3):393–400. doi: 10.1007/s00455-016-9767-x 28013388 10.1007/s00455-016-9767-x

[13] Sia I, Carvajal P, Lacy A, Carnaby G, Crary M. Hyoid and laryngeal excursion kinematics–magnitude, duration and velocity–changes following successful exercise‐based dysphagia rehabilitation: MDTP. J Oral Rehabil. 2015;42(5):331–9. doi: 10.1111/joor.12259 25488830 10.1111/joor.12259

[14] Nagy A, Molfenter SM, Péladeau-Pigeon M, Stokely S, Steele CM. The effect of bolus volume on hyoid kinematics in healthy swallowing. Biomed Res Int. 2014. 10.1155/2014/738971 PMC3981110 24779015

[15] Wang TG, Chang YC, Chen WS, Lin PH, Hsiao TY. Reduction in hyoid bone forward movement in irradiated nasopharyngeal carcinoma patients with dysphagia. Arch Phys Med Rehabil. 2010;91(6):926–31. doi: 10.1016/j.apmr.2010.02.011 20510985 10.1016/j.apmr.2010.02.011

[16] Zhang J, Zhou Y, Wei N, Yang B, Wang A, Zhou H, et al Laryngeal Elevation Velocity and Aspiration in Acute Ischemic Stroke Patients. PloS one. 2016;11(9):e0162257 doi: 10.1371/journal.pone.0162257 27583413 10.1371/journal.pone.0162257 PMC5008618

[17] Steele CM, Bailey GL, Chau T, Molfenter SM, Oshalla M, Waito AA, et al The relationship between hyoid and laryngeal displacement and swallowing impairment. Clin otolaryngol. 2011;36(1):30–6. doi: 10.1111/j.1749-4486.2010.02219.x 21414151 10.1111/j.1749-4486.2010.02219.x PMC3757521

[18] Ludlow CL, Humbert I, Saxon K, Poletto C, Sonies B, Crujido L. Effects of surface electrical stimulation both at rest and during swallowing in chronic pharyngeal dysphagia. Dysphagia. 2007;22(1):1–10. doi: 10.1007/s00455-006-9029-4 16718620 10.1007/s00455-006-9029-4 PMC1790908

[19] Kellen PM, Becker DL, Reinhardt JM, Van Daele DJ. Computer-assisted assessment of hyoid bone motion from videofluoroscopic swallow studies. Dysphagia. 2010;25(4):298–306. doi: 10.1007/s00455-009-9261-9 19856024 10.1007/s00455-009-9261-9

[20] Logemann J. Manual for the videofluoroscopic study of swallowing Austin, TX: Pro-Ed. 1993.

[21] Freeman H. Machine Vision for Three-Dimensional Scenes. 1^st^ ed. Boston: Academic Press 1990.

[22] Michalewicz Z, Hartley SJ. Genetic algorithms+ data structures = evolution programs. Mathematical Intelligencer. 1996;18(3):71.

[23] Potratz JR, Dengel G, Robbins J. A comparison of swallowing in three subjects using an interactive image processing system. Proceedings Fifth Annual IEEE Symposium on Computer-Based Medical Systems, Durham, NC, 1992, pp. 115–122.

[24] Kim YH, Oh BM, Jung IY, Lee JC, Lee GJ, Han TR. Spatiotemporal characteristics of swallowing in Parkinson's disease. Laryngoscope. 2015;125(2):389–95. doi: 10.1002/lary.24869 25093527 10.1002/lary.24869

[25] Yabunaka K, Sanada H, Sanada S, Konishi H, Hashimoto T, Yatake H, et al Sonographic assessment of hyoid bone movement during swallowing: a study of normal adults with advancing age. Radiol Phys and Technol. 2011;4(1):73–7. 10.1007/s12194-010-0107-9 20945118

[26] Chen Y, Barron JL, Taves DH, Martin RE. Computer measurement of oral movement in swallowing. Dysphagia. 2001;16(2):97–109. doi: 10.1007/PL00021292 11305227 10.1007/PL00021292

[27] Steele CM, Cichero JA. Physiological factors related to aspiration risk: a systematic review. Dysphagia. 2014;29(3):295–304. doi: 10.1007/s00455-014-9516-y 24562507 10.1007/s00455-014-9516-y PMC4062811

[28] Lee JC, Seo HG, Lee WH, Kim HC, Han TR, Oh BM. Computer-assisted detection of swallowing difficulty. Comput Methods Programs Biomed. 2016;134:79–88. doi: 10.1016/j.cmpb.2016.07.010 27480734 10.1016/j.cmpb.2016.07.010

[29] van der Kruis JG, Baijens LW, Speyer R, Zwijnenberg I. Biomechanical analysis of hyoid bone displacement in videofluoroscopy: a systematic review of intervention effects. Dysphagia. 2011;26(2):171–82. doi: 10.1007/s00455-010-9318-9 21165750 10.1007/s00455-010-9318-9 PMC3098989

[30] Shi JQ, Choi T. Gaussian process regression analysis for functional data Boca Raton, FL: CRE press 2011.

[31] Aljabar P, Heckemann RA, Hammers A, Hajnal JV, Rueckert D. Multi-atlas based segmentation of brain images: atlas selection and its effect on accuracy. Neuroimage. 2009;46(3):726–38. doi: 10.1016/j.neuroimage.2009.02.018 19245840 10.1016/j.neuroimage.2009.02.018

[32] Chupin M, Gérardin E, Cuingnet R, Boutet C, Lemieux L, Lehéricy S, et al Fully automatic hippocampus segmentation and classification in Alzheimer’s disease and mild cognitive impairment applied on data from ADNI. Hippocampus. 2009;19(6):579 doi: 10.1002/hipo.20626 19437497 10.1002/hipo.20626 PMC2837195

[33] Lazareck LJ, Moussavi ZM. Classification of normal and dysphagic swallows by acoustical means. IEEE Trans Biomed Eng. 2004;51(12):2103–12. doi: 10.1109/TBME.2004.836504 15605857 10.1109/TBME.2004.836504

[34] Lee J, Steele CM, Chau T. Classification of healthy and abnormal swallows based on accelerometry and nasal airflow signals. Artif Intell Med. 2011;52(1):17–25. doi: 10.1016/j.artmed.2011.03.002 21549579 10.1016/j.artmed.2011.03.002

[35] Marron JS, Ramsay JO, Sangalli LM, Srivastava A. Functional data analysis of amplitude and phase variation. Statistical Science. 2015;30(4):468–84.

[36] Sangalli LM, Secchi P, Vantini S, Veneziani A. A case study in exploratory functional data analysis: geometrical features of the internal carotid artery. Journal of the American Statistical Association. 2009;104(485):37–48.

[37] Srivastava A, Klassen E, Joshi SH, Jermyn IH. Shape analysis of elastic curves in Euclidean spaces. IEEE Transactions on Pattern Analysis and Machine Intelligence. 2011;33(7):1415–28. doi: 10.1109/TPAMI.2010.184 20921581 10.1109/TPAMI.2010.184

