# Supplementary material for: Semi-automatic tracking, smoothing and segmentation of hyoid bone motion from videofluoroscopic swallowing study
Source: PLoS One. 2017 Nov 28;12(11):e0188684. doi: 10.1371/journal.pone.0188684 (PMC5705154; doi:10.1371/journal.pone.0188684)
Supplement: S1 File — (DOCX) [file pone.0188684.s001.docx]

**S1 File**

1. **Validation results**

**Partly-masked group (10 circles from 9 trajectories)**


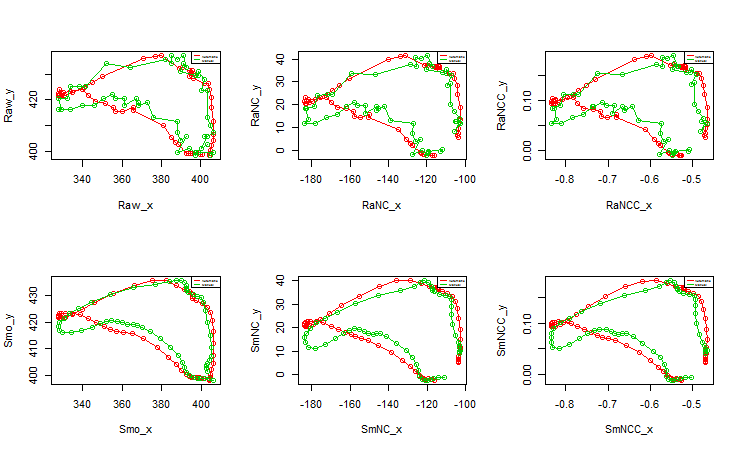


"aspiration(+)_No.3(1-230)"


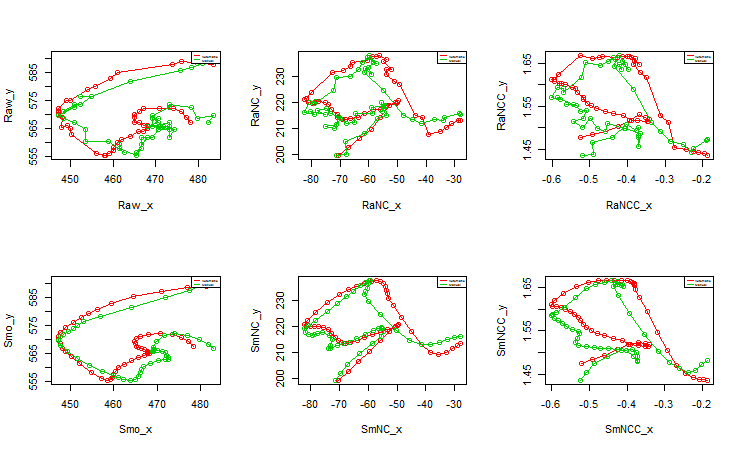


"aspiration(-)_No.10(1-300)"


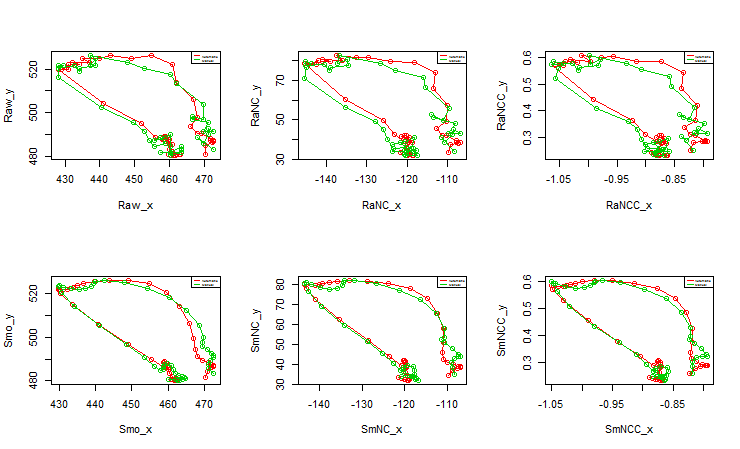


"aspiration(-)_No.11(1-325)"


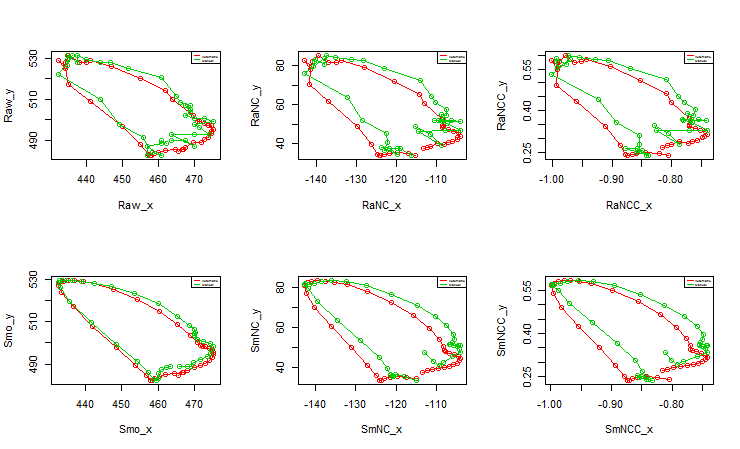


"aspiration(-)_No.11(500-800)"


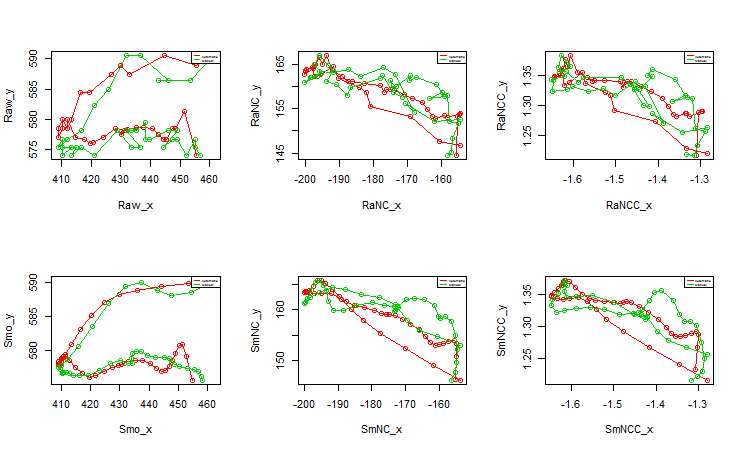


"aspiration(-)_No.12(1-210)"


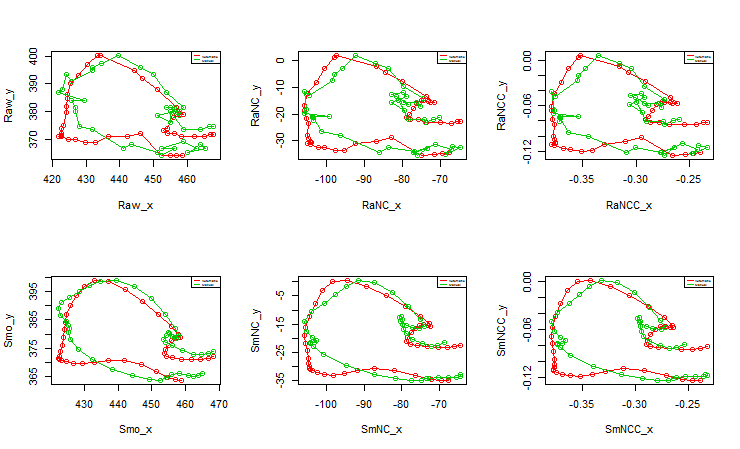


"aspiration(+)_No.16(1-300)" ---(73 125)


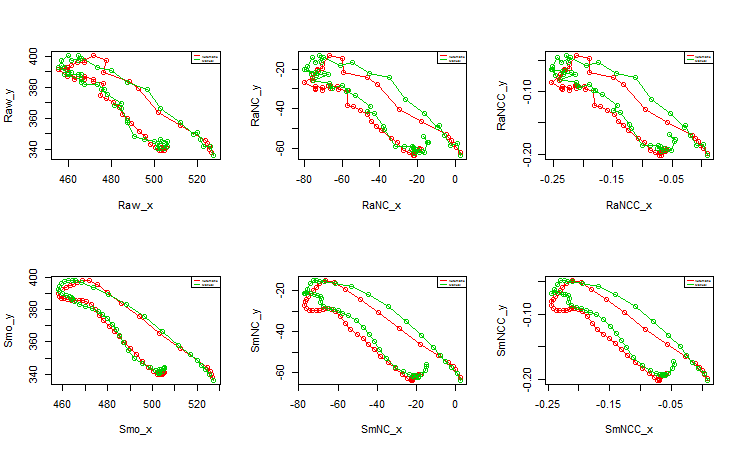


"aspiration(+)_No.16(1-300)" ---(215 260)


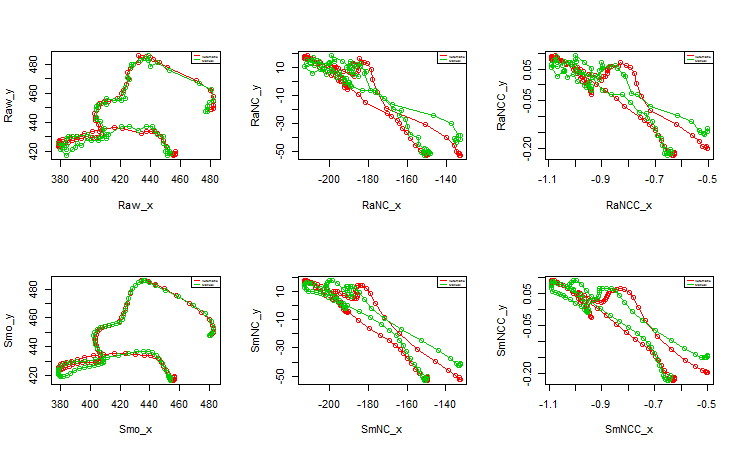


"aspiration(-)_No.18(261-486)"


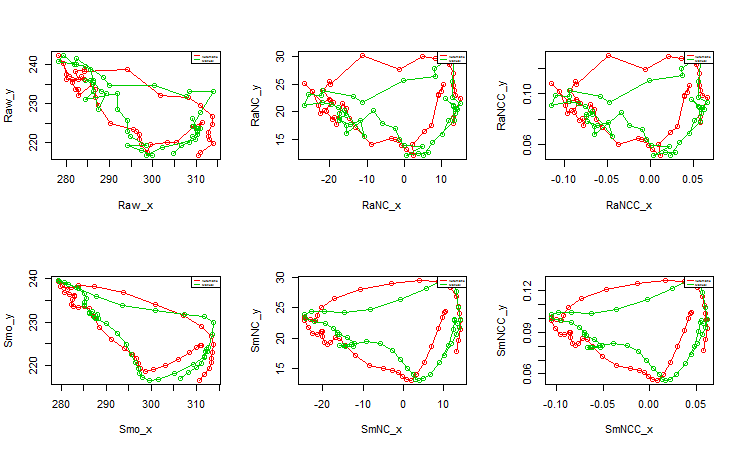


"aspiration(-)_No.23(1-430)"


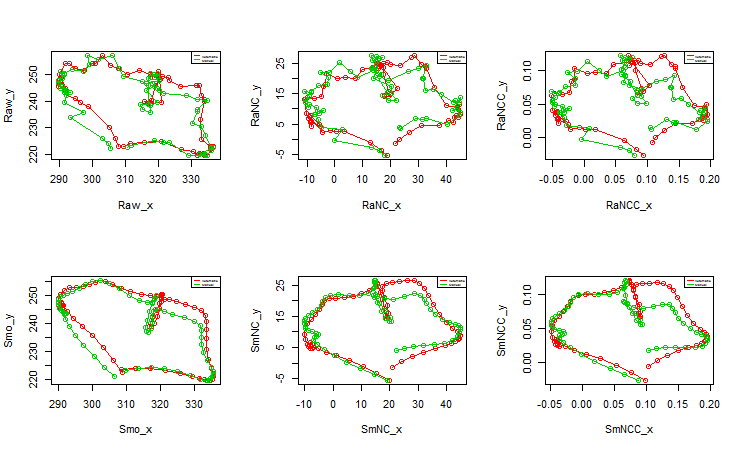


"aspiration(-)_No.27(275-460)"

**Unmasked group (10 circles from 8 trajectories)**
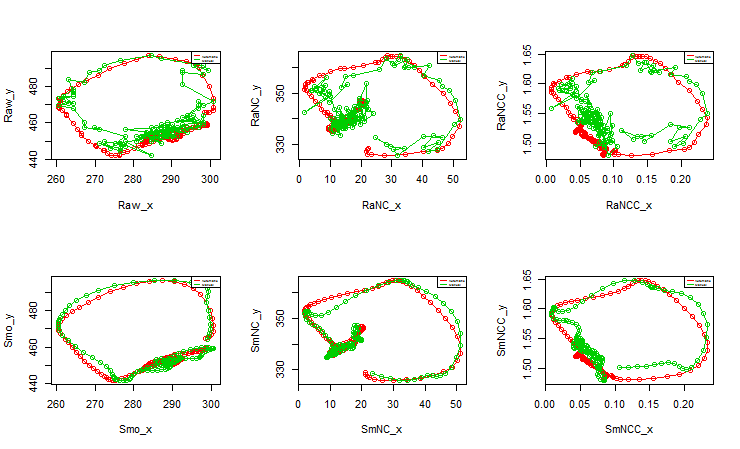


"aspiration(+)_No.2(1-280)"


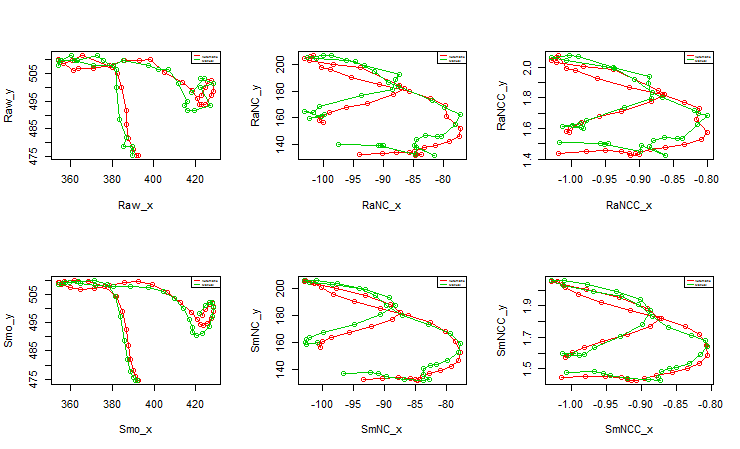


"aspiration(-)_No.5(1-250)"---(190 225)


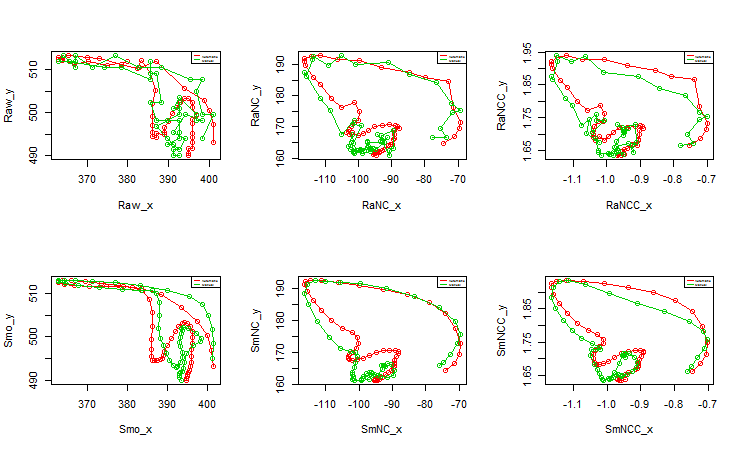


"aspiration(-)_No.6(171-320)"


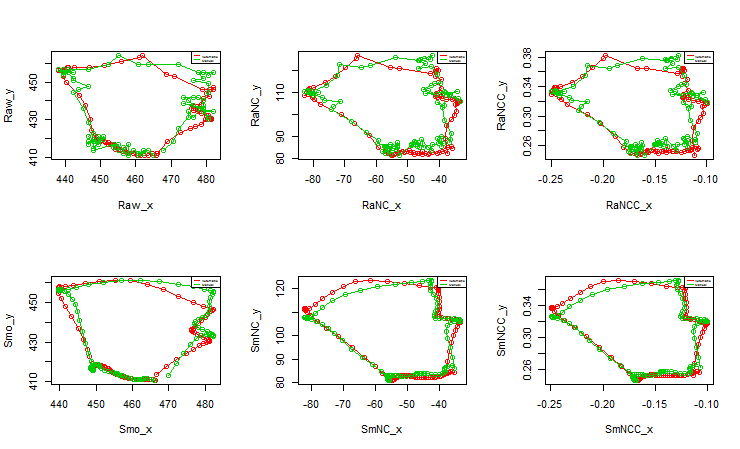


"aspiration(-)_No.7(1-200)"


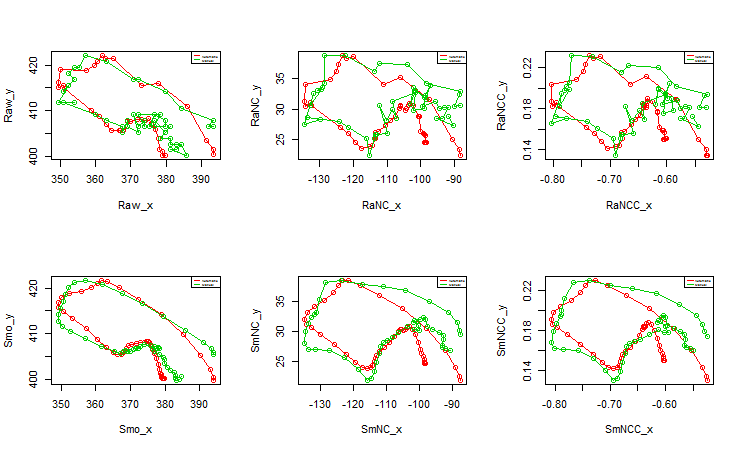


"aspiration(-)_No.8(151-295)"


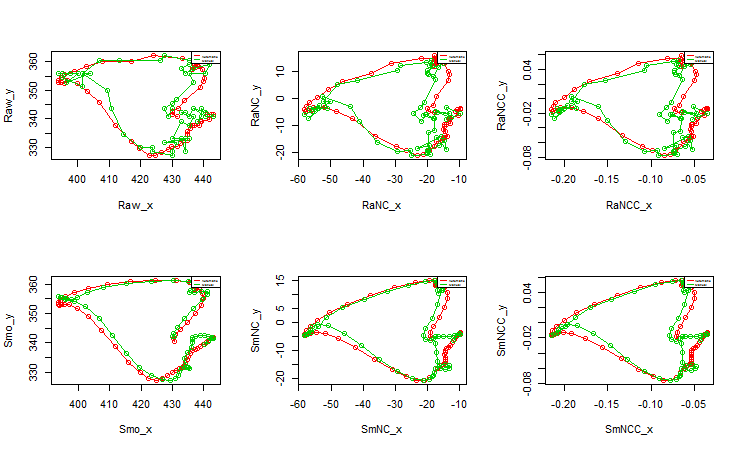


"aspiration(-)_No.9(1-130)"


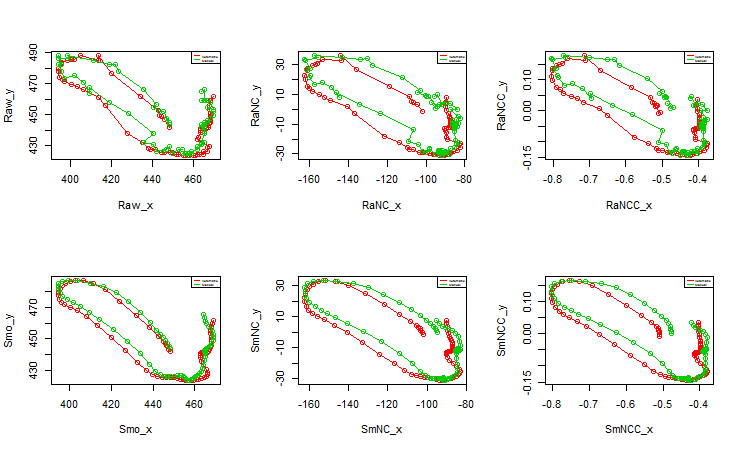


"aspiration(-)_No.13(555-728)"


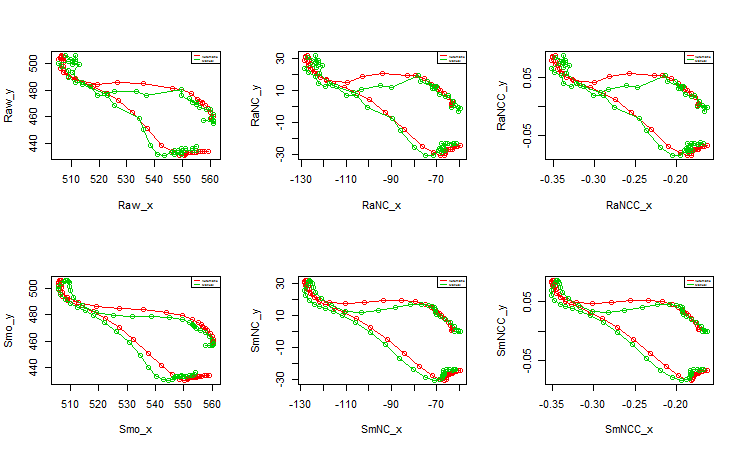


"aspiration(+)_No.14(1-190)"


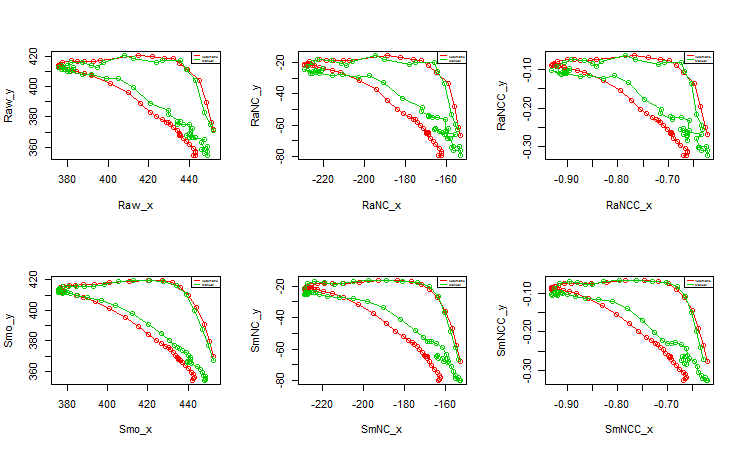


"aspiration(+)_No.17(1-200)"


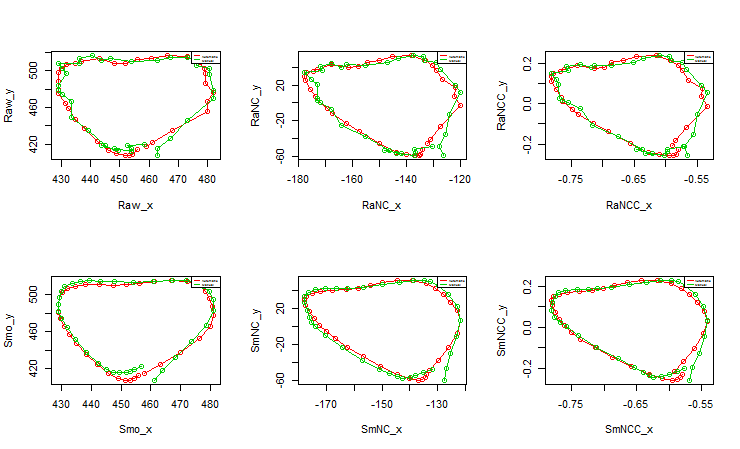


"aspiration(-)_No.5(1-250)" ---(1 55)

1. **Automatic segmentation results**

**Non-aspiration group (n=19)**


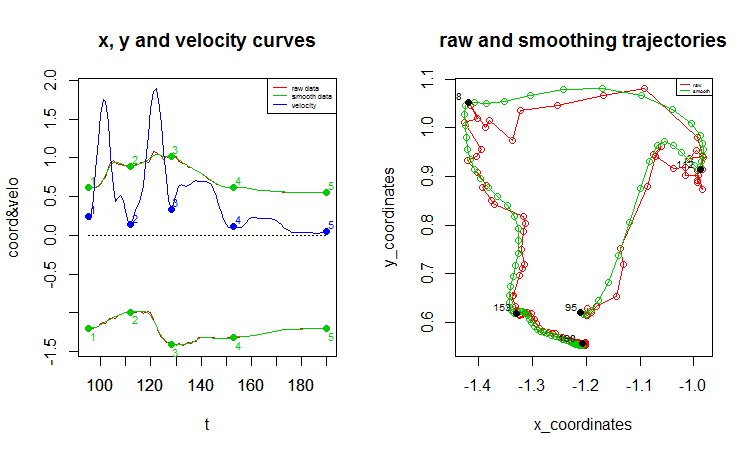


"aspiration(-)_No.1(45-190)"


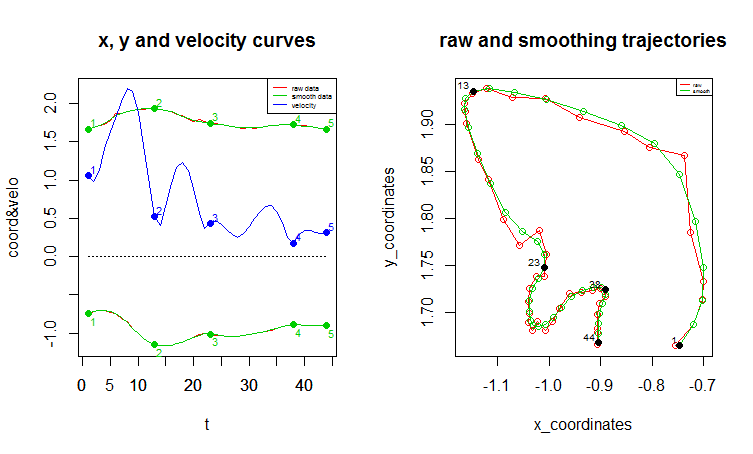


"aspiration(-)_No.5(1-250)"


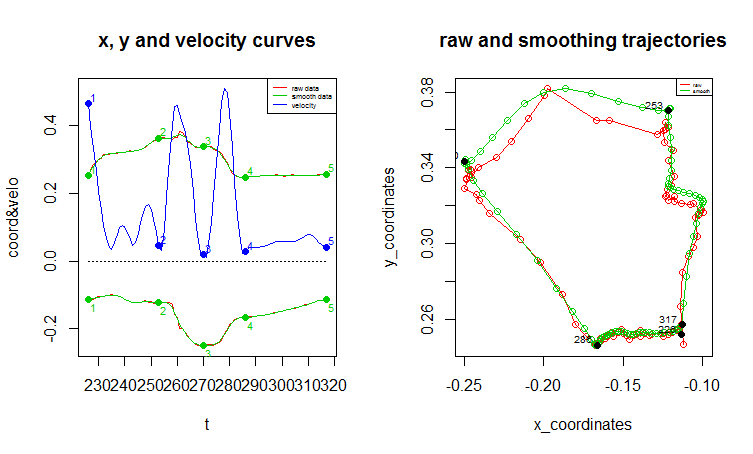


"aspiration(-)_No.6(171-320)"


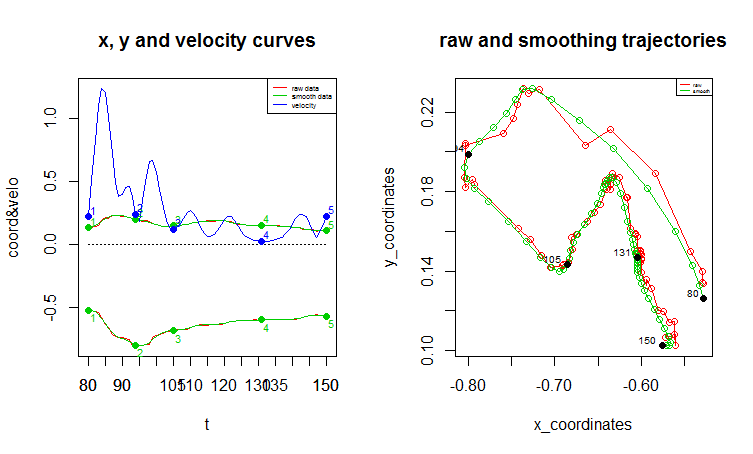


"aspiration(-)_No.7(1-200)"


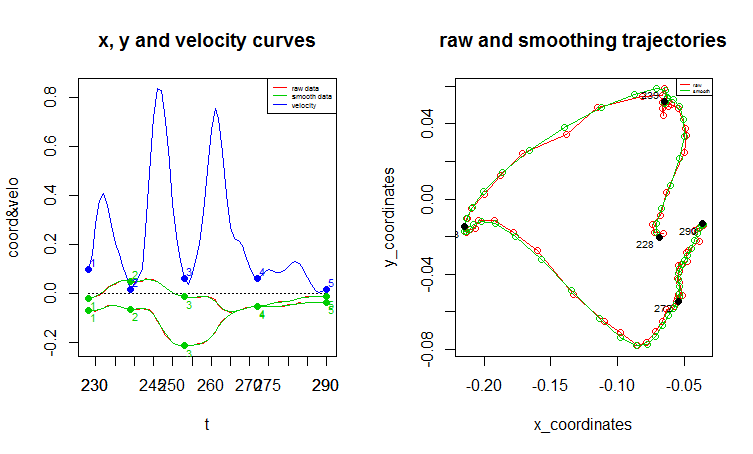


"aspiration(-)_No.8(151-295)"


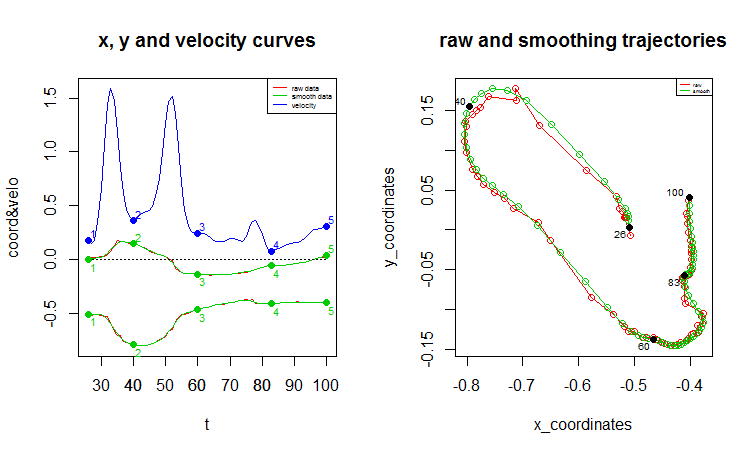


"aspiration(-)_No.9(1-130)"


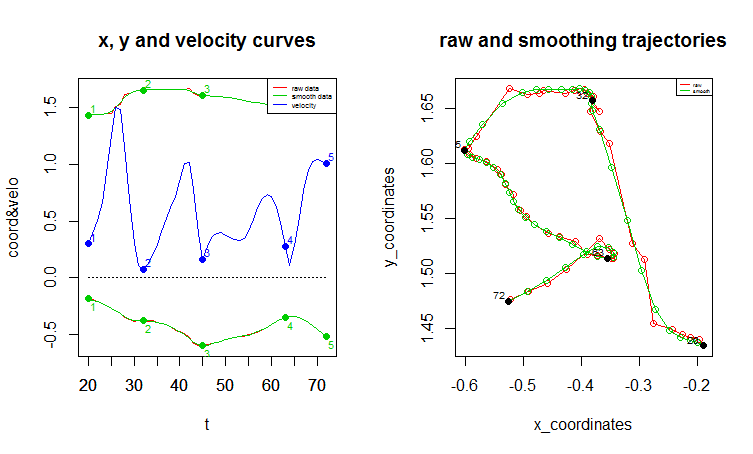


"aspiration(-)_No.10(1-300)"


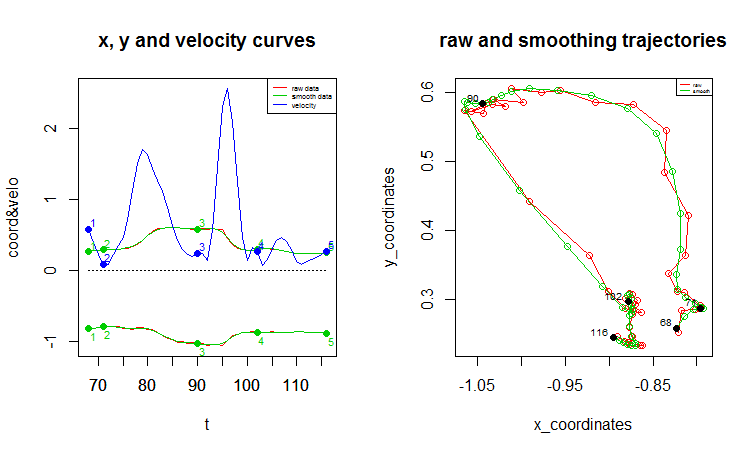


"aspiration(-)_No.11(1-325)"


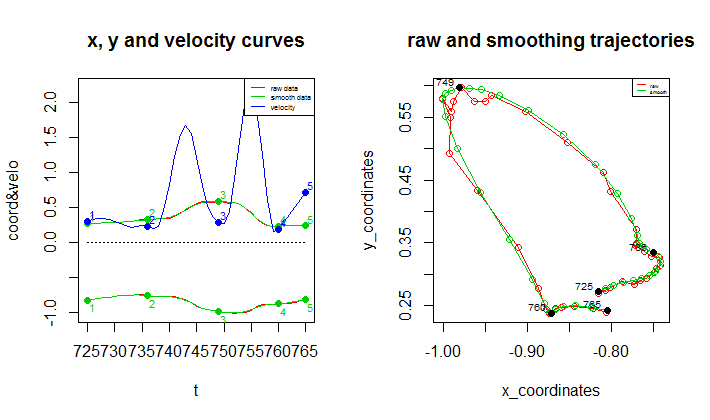


"aspiration(-)_No.11(500-800)"


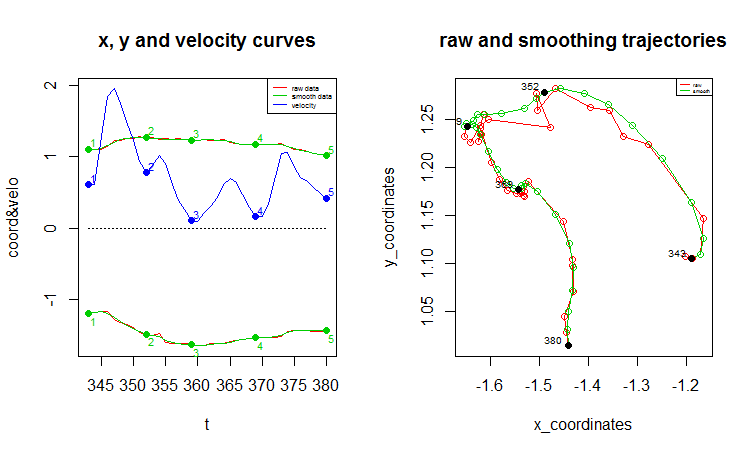


"aspiration(-)_No.12(211-387)"


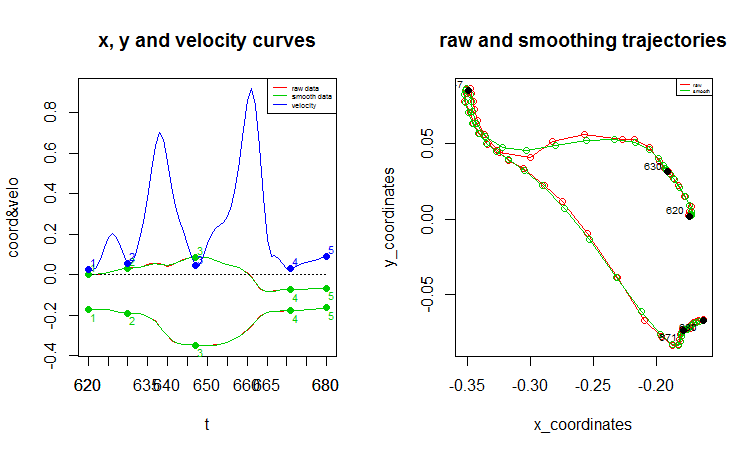


"aspiration(-)_No.13(555-728)"


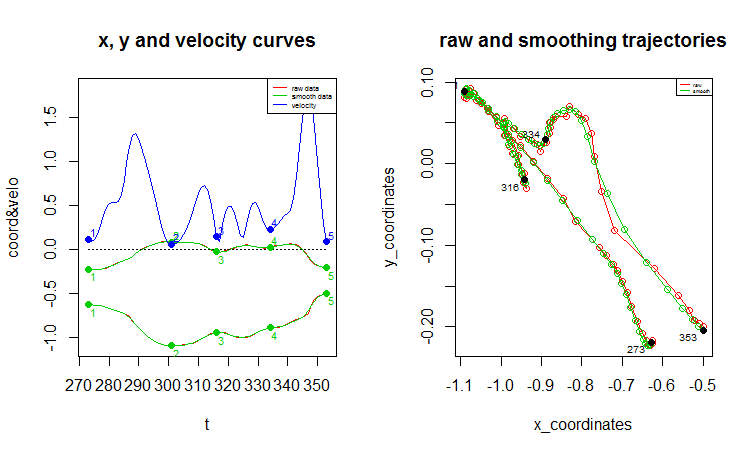


"aspiration(-)_No.18(261-486)"


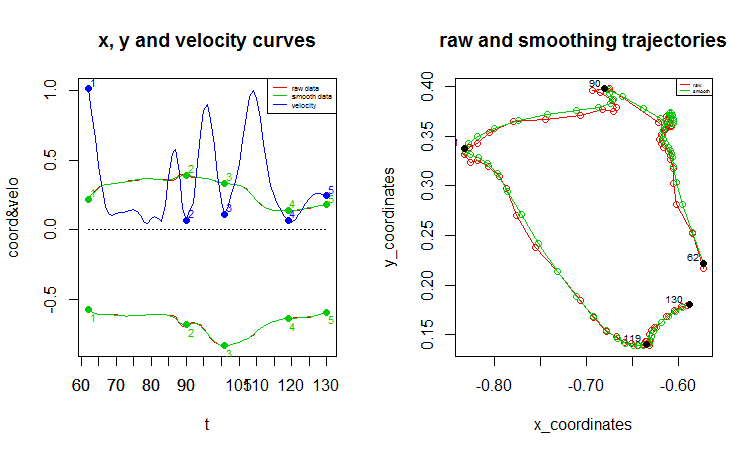


"aspiration(-)_No.19(1-250)"


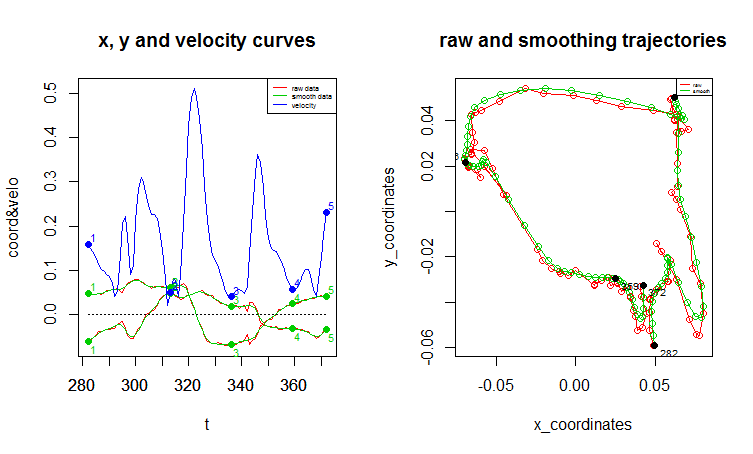


"aspiration(-)_No.21(1-621)"


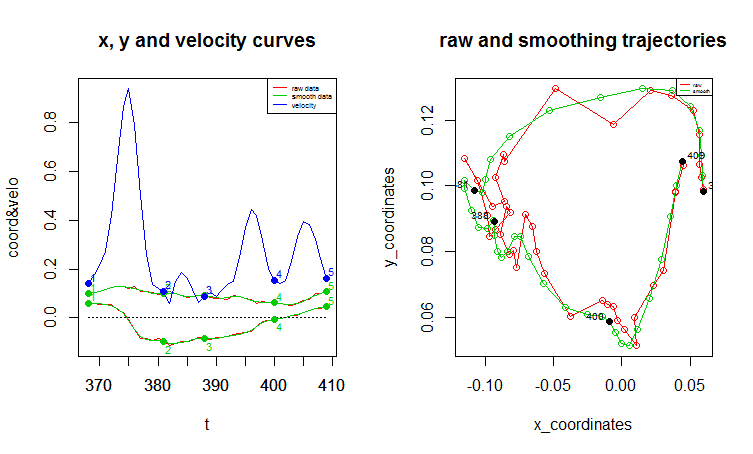


"aspiration(-)_No.23(1-430)"


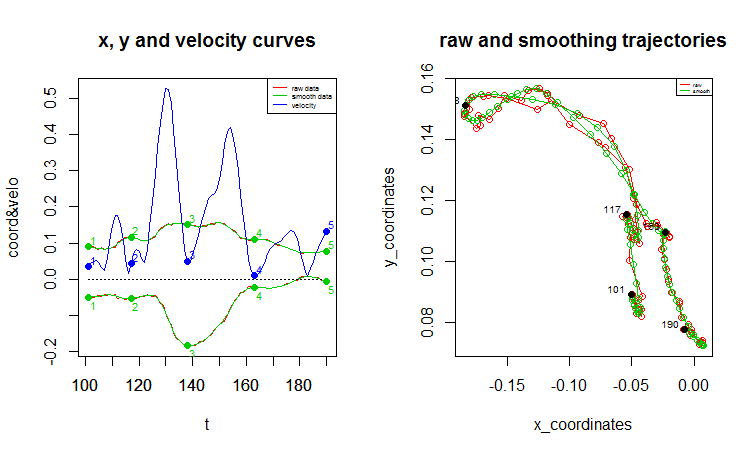


"aspiration(-)_No.26(1-821)"


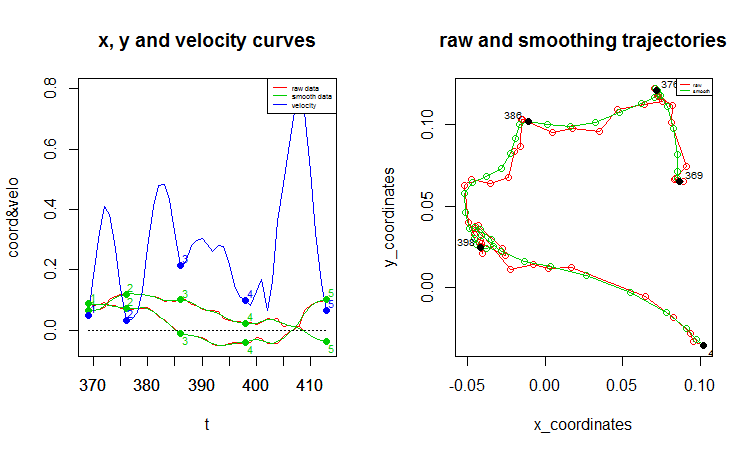


"aspiration(-)_No.27(275-460)"


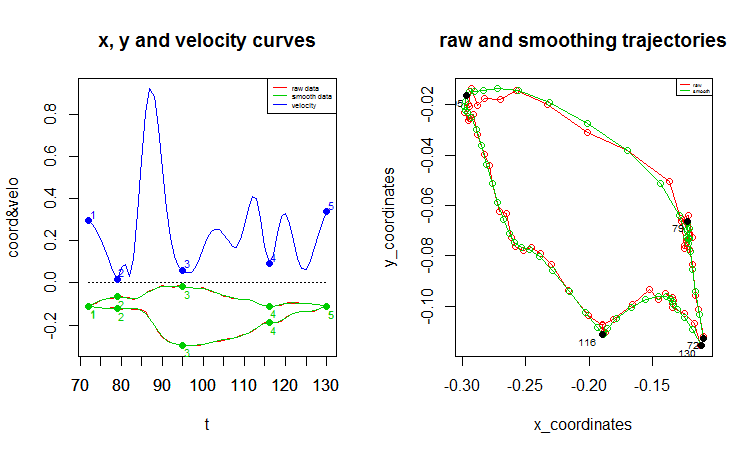


"aspiration(-)_No.28(1-583)"


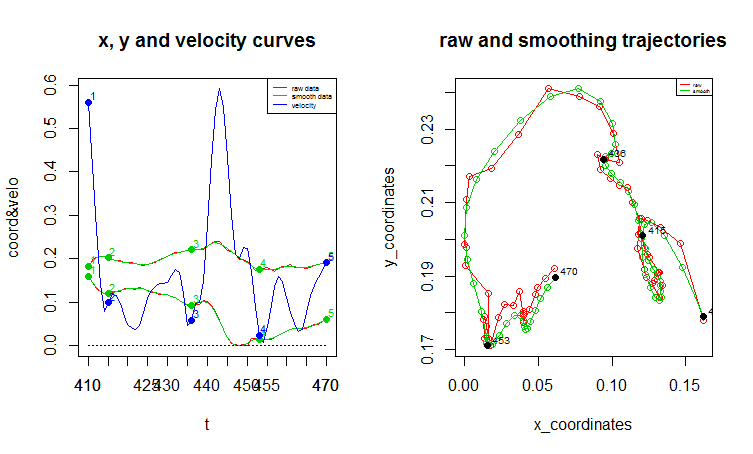


"aspiration(-)_No.29(1-525)"


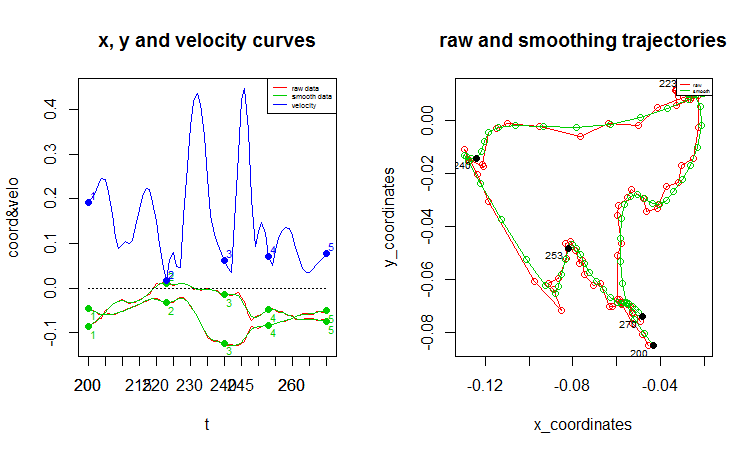


"aspiration(-)_No.30(1-618)"

Aspiration group (n=12)


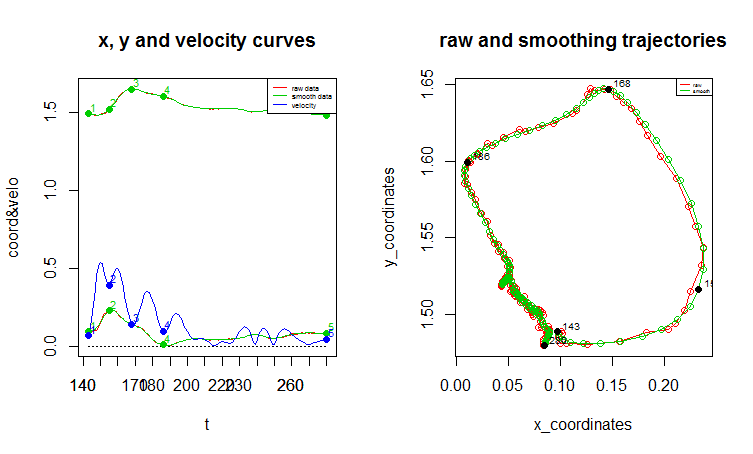


"aspiration(+)_No.2(1-280)"


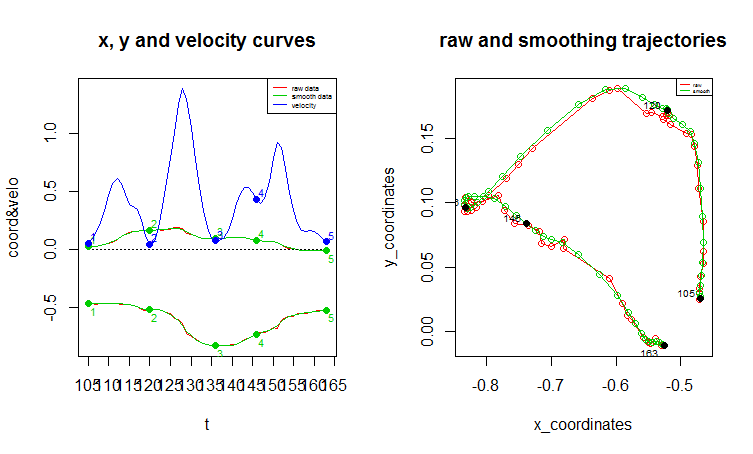


"aspiration(+)_No.3(1-230)"


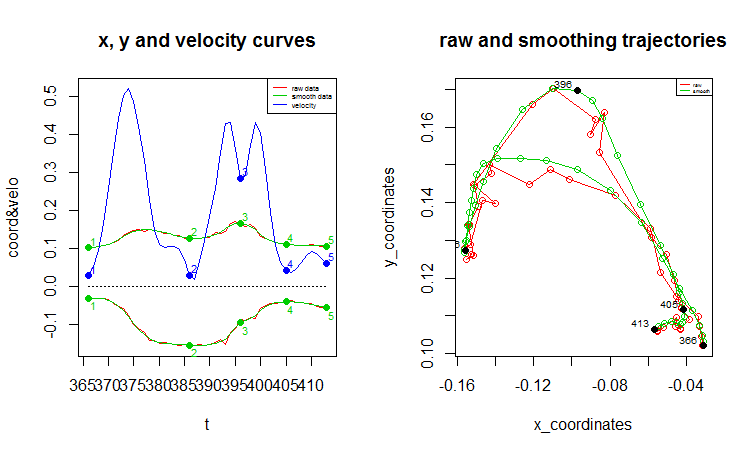


"aspiration(+)_No.4(271-430)"


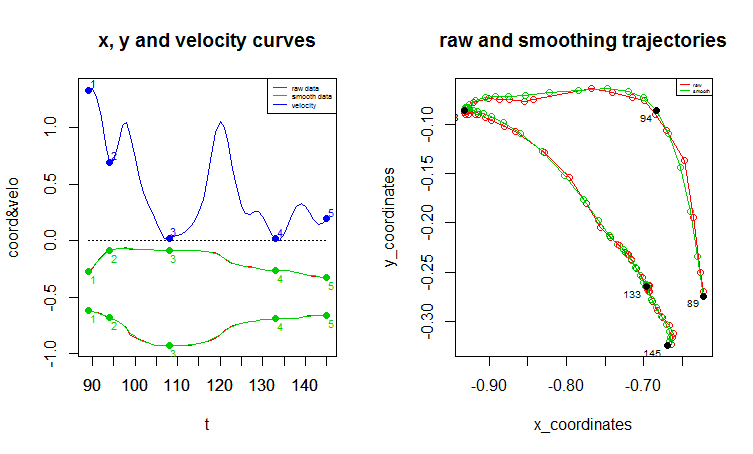


"aspiration(+)_No.14(1-190)"


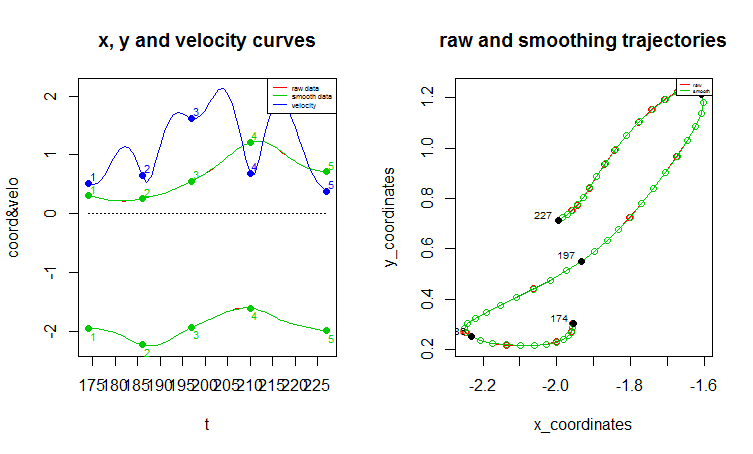


"aspiration(+)_No.15(1-300)"


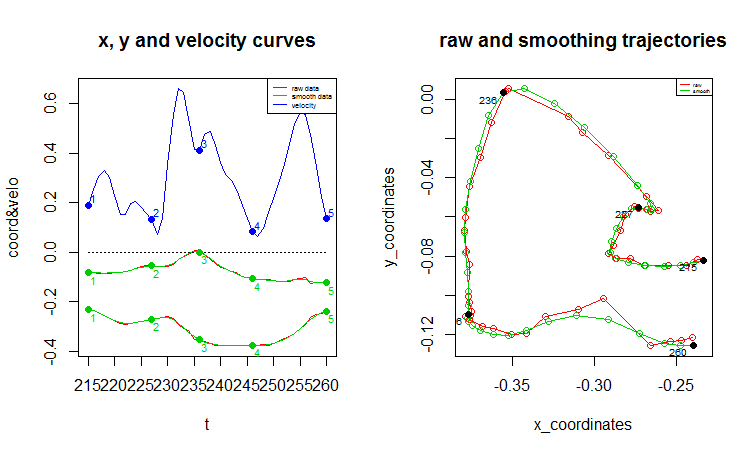


"aspiration(+)_No.16(1-300)" (two totally different segmentations)


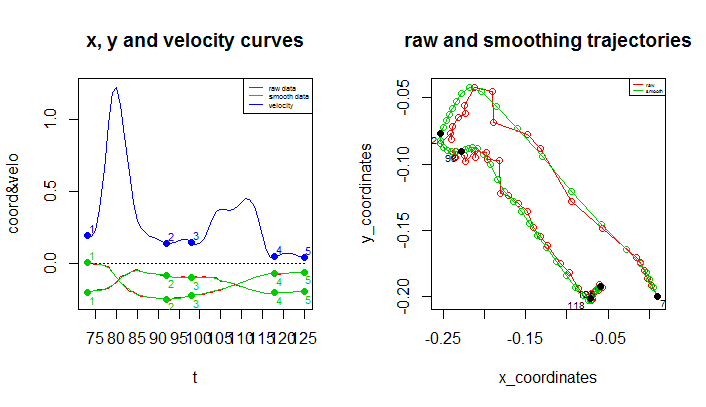


"aspiration(+)_No.16(1-300)"


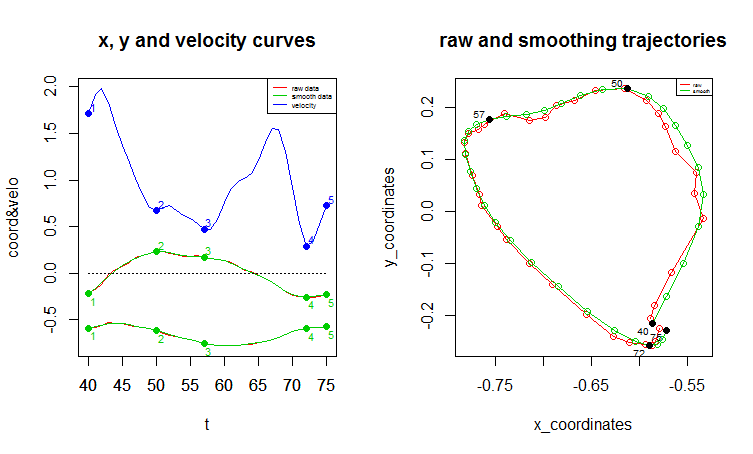


"aspiration(+)_No.17(1-200)"


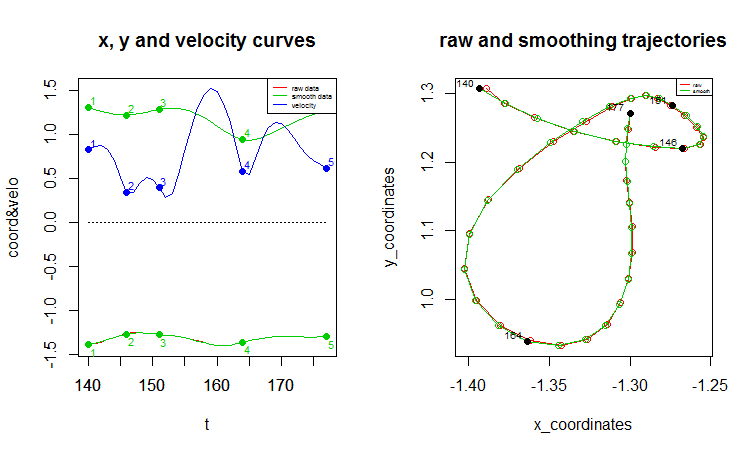


"aspiration(+)_No.20(1-229)"


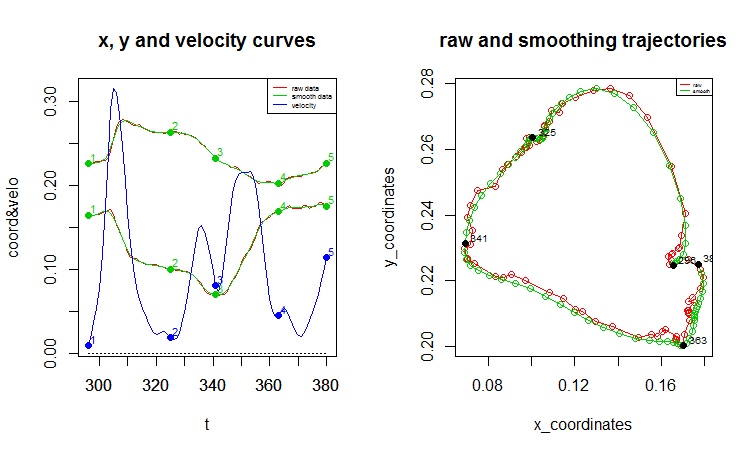


"aspiration(+)_No.22(100-531)"


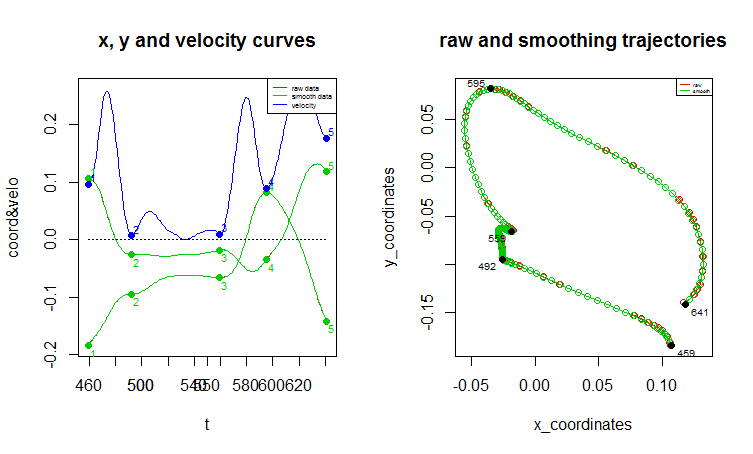


"aspiration(+)_No.24(1-774)"


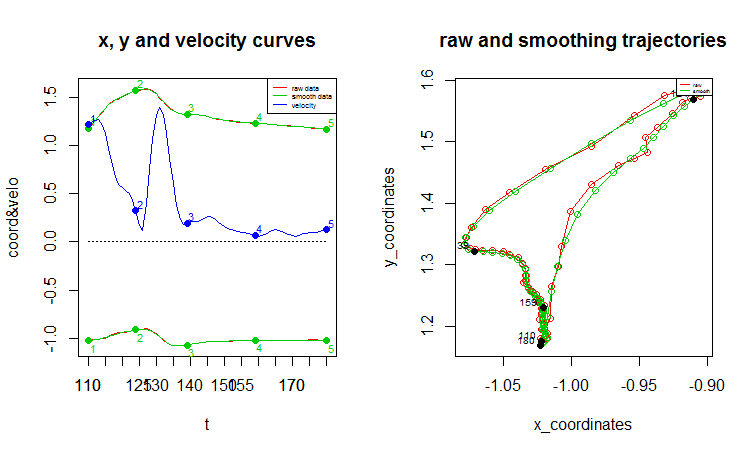


"aspiration(+)_No.31(1-300)"
