# Supplementary material for: Semi-automatic tracking, smoothing and segmentation of hyoid bone motion from videofluoroscopic swallowing study
Source: PLoS One. 2017 Nov 28;12(11):e0188684. doi: 10.1371/journal.pone.0188684 (PMC5705154; doi:10.1371/journal.pone.0188684)
Supplement: S2 File — (ZIP) [file pone.0188684.s002.zip › Explanations for the data in this folder.docx]

**The raw data included in the folder named “Data_Semi_Automatic_Tracking”**

Those data were obtained by our developed software. For each video clip, there are at least one pair of recorded txt files, with one recording coordinates of red crosses in C2 and C4 (Fig 3) and the other saving the coordinates of hyoid bone, frame by frame.

Take the No.1 video clip for example, "1_axis_fr45to190.txt" recorded the (x,y) coordinates of red crosses in C2 and C4, from 45th frame to 190th frame. "1_hyoid_fr45to190.txt" recorded the (x,y) coordinates of the hyoid bone, from 45th frame to 190th frame.

Some of the video clips have more than one pairs of txt files, each of which generally represents one rough circle corresponding to the hyoid bone movement of one swallow. As for No.15 video clip, it is too difficult to track by software, so that we use the alternative manual tracking method.
