# Supplementary material for: Semi-automatic tracking, smoothing and segmentation of hyoid bone motion from videofluoroscopic swallowing study
Source: PLoS One. 2017 Nov 28;12(11):e0188684. doi: 10.1371/journal.pone.0188684 (PMC5705154; doi:10.1371/journal.pone.0188684)
Supplement: S3 File — (ZIP) [file pone.0188684.s003.zip › Explanations for the data in this folder.docx]

**The raw data included in the folder named “Data_Manual_Tracking”**

These data were tracked by trained observer. For instance, "1_manualtracking_fr45to190" saved the coordinates of C2, C4 and hyoid bone, from 45th frame to 190th frame.
