# Supplementary material for: Semi-automatic tracking, smoothing and segmentation of hyoid bone motion from videofluoroscopic swallowing study
Source: PLoS One. 2017 Nov 28;12(11):e0188684. doi: 10.1371/journal.pone.0188684 (PMC5705154; doi:10.1371/journal.pone.0188684)
Supplement: S4 File — (ZIP) [file pone.0188684.s004.zip › Explanations for the data in this folder.docx]

**The raw data included in the folder called “Data_Manual_Interrater_Reliability”**

These data were manually tracked by two independent trained observer. For instance, "2_manualtracking_fr1to280" is the record of the first observer’s tracking while "2_manualtracking0_fr1to280" is from another one.
